# Significant alterations of intestinal symbiotic microbiota induced by intraperitoneal vaccination mediate changes in intestinal metabolism of NEW Genetically Improved Farmed Tilapia (NEW GIFT, *Oreochromis niloticus*)

**DOI:** 10.1186/s40168-022-01409-6

**Published:** 2022-12-12

**Authors:** Zhenbing Wu, Qianqian Zhang, Jicheng Yang, Jinyong Zhang, Jie Fu, Chenyuan Dang, Mansen Liu, Shuyi Wang, Yaoyao Lin, Jingwen Hao, Meiqi Weng, Derong Xie, Aihua Li

**Affiliations:** 1 State Key Laboratory of Freshwater Ecology and Biotechnology, Institute of Hydrobiology, Chinese Academy of Sciences, Wuhan, 430072 China; 2 School of Environmental Science and Engineering, Huazhong University of Science and Technology, Wuhan, 430074 China; 3 Key Laboratory of Aquaculture Disease Control, Ministry of Agriculture, Beijing, China; 4 National Aquatic Biological Resource Center, NABRC, Wuhan, 430072 China; 5 College of Fisheries and Life, Dalian Ocean University, Dalian, 116023 China; 6 Laboratory of Aquatic Parasitology, School of Marine Science and Engineering, Qingdao Agricultural University, Qingdao, 266237 China; 7 College of Advanced Agricultural Sciences, University of Chinese Academy of Sciences, Beijing, 100049 China

**Keywords:** Nile tilapia, Inactivated vaccination, Symbiotic microbiota, High-throughput sequencing, Intestinal metabolism

## Abstract

**Background:**

After millions of years of coevolution, symbiotic microbiota has become an integral part of the host and plays an important role in host immunity, metabolism, and health. Vaccination, as an effective means of preventing infectious diseases, has been playing a vital role in the prevention and control of human and animal diseases for decades. However, so far, minimal is known about the effect of vaccination on fish symbiotic microbiota, especially mucosal microbiota, and its correlation with intestinal metabolism remains unclear.

**Methods:**

Here we reported the effect of an inactivated bivalent *Aeromonas hydrophila*/*Aeromonas veronii* vaccine on the symbiotic microbiota and its correlation with the intestinal metabolism of farmed adult Nile tilapia (*Oreochromis niloticus*) by 16S rRNA gene high-throughput sequencing and gas chromatography-mass spectrometry metabolomics.

**Results:**

Results showed that vaccination significantly changed the structure, composition, and predictive function of intestinal mucosal microbiota but did not significantly affect the symbiotic microbiota of other sites including gill mucosae, stomach contents, and stomach mucosae. Moreover, vaccination significantly reduced the relative abundance values of potential opportunistic pathogens such as *Aeromonas*, *Escherichia*–*Shigella*, and *Acinetobacter* in intestinal mucosae. Combined with the enhancement of immune function after vaccination, inactivated bivalent *Aeromonas* vaccination had a protective effect against the intestinal pathogen infection of tilapia. In addition, the metabolite differential analysis showed that vaccination significantly increased the concentrations of carbohydrate-related metabolites such as lactic acid, succinic acid, and gluconic acid but significantly decreased the concentrations of multiple lipid-related metabolites in tilapia intestines. Vaccination affected the intestinal metabolism of tilapia, which was further verified by the predictive function of intestinal microbiota. Furthermore, the correlation analyses showed that most of the intestinal differential microorganisms were significantly correlated with intestinal differential metabolites after vaccination, confirming that the effect of vaccination on intestinal metabolism was closely related to the intestinal microbiota.

**Conclusions:**

In conclusion, this paper revealed the microbial and metabolic responses induced by inactivated vaccination, suggesting that intestinal microbiota might mediate the effect of vaccination on the intestinal metabolism of tilapia. It expanded the novel understanding of vaccine protective mechanisms from microbial and metabolic perspectives, providing important implications for the potential influence of vaccination on human intestinal microbiota and metabolism.

Video Abstract

**Supplementary Information:**

The online version contains supplementary material available at 10.1186/s40168-022-01409-6.

## Background

Currently, the ongoing, recurring coronavirus disease 2019 (COVID-19) pandemic poses serious threats to global public health [1]. COVID-19 vaccination is an effective means of epidemic prevention and control, which can successfully reduce overall morbidity and mortality [2]. Vaccination is the safest, most reliable means of preventing human and animal infectious diseases [3]. As an effective method of preventing a wide range of bacterial and viral diseases, vaccination has been playing a key role in aquaculture disease control for decades, contributing to its environmental, social, and economic sustainability [4]. In particular, vaccines based on inactivated bacterial pathogens have proven highly effective for fish [5]. The whole-cell inactivated vaccine has been widely used to prevent and control bacterial diseases in aquaculture due to the advantages of safety, simple preparation, and easy preservation [4].

Over the past 30 years, with the rapid growth and highly intensive development of the aquaculture industry, aquaculture animals have been threatened by serious diseases caused by viruses, bacteria, fungi, parasites, or other undiagnosed, emerging pathogens [6]. Among them, one of the major threats that limit the sustainable development of aquaculture is the economic losses imposed by the high mortality in farmed animals resulting from the outbreak of bacterial infectious diseases [4]. Nile tilapia (*Oreochromis niloticus*) is an important aquaculture species in China, accounting for over 50% of the world’s total tilapia production [7]. However, in recent years, the frequent outbreaks of various bacterial infectious diseases are considered primary constraints and continuous challenges to the growth of the tilapia industry [8]. Numerous studies of bacterial diseases of tilapia have been reported so far, such as infectious diseases caused by *Flavobacterium columnare* [9], *Aeromonas* species [10, 11], and *Streptococcus* [12, 13]. The *Aeromonas* species such as *Aeromonas hydrophila* and *Aeromonas veronii* are ubiquitous pathogens, which can cause hemorrhage disease, ulceration syndrome, and motile aeromonad septicemia in fish [14]. For a long time, antibiotics and antimicrobial agents were mainly used in aquaculture to protect fish from bacterial infection [15]. However, the abuse of antibiotics not only greatly accelerates the generation of bacterial resistance but also brings about the problem of antibiotic residue, causing serious food safety and public environmental health risks [15, 16]. To reduce the use of antibiotics, vaccination has been widely used in aquaculture to prevent infectious diseases [4, 5]. At present, a large number of inactivated whole bacteria vaccines have been developed and applied against infections of various pathogens, including *Vibrio*, *A*. *hydrophila*, *Streptococcus*, and other common pathogens [4].

The three major infection sites of fish are the skin, gills, and gastrointestinal tract, which are inhabited by complex microbial communities [17]. After millions of years of coevolution, these symbiotic microbiotas have become an integral component of fish and play critical roles in fish health [18, 19]. Notably, the intestinal microbiota of fish is involved in many vital physiological processes such as contributing to digestion and metabolism, stimulating the development of the immune system, and preventing the attachment and proliferation of opportunistic pathogens [20]. Conversely, the dysbiosis of fish intestinal microbiota is closely associated with the outbreaks of severe diseases [21, 22]. In recent years, many studies have shown that fish intestinal microbiota is closely related to intestinal mucosal immunity [23, 24]. Fish intestinal microbiota directly or indirectly regulates intestinal mucosal immunity through the action of barrier cells, whereas intestinal mucosal immunity, in turn, controls the composition of intestinal microbiota [24, 25]. Given the ability of vaccines to enhance immune function, the fish intestinal microbiota is likely an important, underappreciated factor in vaccine development.

Vaccines contribute to inducing antigen-specific systemic and mucosal immunity, whereas intestinal microbiota affects the immune-inducing effect of vaccines [26, 27]. The correlation between human symbiotic microbiota and vaccine efficacy has been widely reported. The microbial composition of human nasopharynx is affected by influenza virus type and vaccination status [28]. Moreover, intestinal dysbiosis can hinder the effectiveness of vaccines [29, 30]. Therefore, probiotics can improve vaccine effectiveness by regulating the intestinal microbiota [31, 32]. In recent years, several studies have focused on the correlation between fish intestinal microbiota and vaccine efficacy [33–35]. As reported previously, a new recombinant *A*. *hydrophila* vaccine could induce protective immunity of juvenile grass carp against *A. hydrophila* infection through the immune response of tissues, which was closely related to intestinal microbiota [33]. A recent study showed that the diversity and composition of intestinal microbiota especially in the hindgut were significantly altered by the oral *Vibrio mimicus* double-targeted DNA vaccine in grass carp (*Ctenopharyngodon idella*) [35]. The above study also found a correlation between the intestinal microbiota of grass carp and the innate immunity of intestinal mucosa, which was affected by the double-targeted DNA vaccine [35]. Concerning tilapia, a previous study found that the oral administration of attenuated *Streptococcus agalactiae* vaccine altered the diversity and composition of intestinal microbiota in tilapia, but these changes were recoverable [34]. These studies demonstrated close correlations between fish intestinal microbiota and vaccine effectiveness. Consequently, the role of fish symbiotic microbiota in maintaining vaccine effectiveness has attracted increasing attention.

However, to date, minimal information is known about the influence mechanism of vaccination on fish symbiotic microbiota, especially mucosa-associated microbiota. Additionally, the effect of vaccination on fish intestinal metabolism and its correlation with intestinal microbiota remains unexplored. In this paper, the effects of an inactivated bivalent *Aeromonas* vaccine on the structure, composition, and function of the gill, gastrointestinal mucosa-associated, and digesta-associated microbiota in NEW Genetically Improved Farmed Tilapia (NEW GIFT, *O*. *niloticus*) were investigated for the first time by 16S rRNA gene high-throughput sequencing and gas chromatography-mass spectrometry (GC-MS) metabolomics. The effect of vaccination on the immune and metabolic functions of tilapia was further evaluated, and the correlation between altered intestinal microbiota and intestinal metabolism was explored. These results revealed the microbial and metabolic responses of tilapia to vaccination, providing a new perspective for understanding the vaccine protective mechanism. In the context of the current epidemic, important implications for the effects of vaccination on human symbiotic microbiota and metabolic function were provided.

## Materials and methods

### Vaccine preparation

*A*. *hydrophila* and *A*. *veronii* used in the paper were obtained from the blood of naturally diseased fish on August 1, 2006, and maintained at the National Aquatic Biological Resource Center, Institute of Hydrobiology, Chinese Academy of Sciences, Wuhan, China. The pure colonies of *A*. *hydrophila* and *A*. *veronii* were cultured and isolated by tryptic soy agar (TSA; Becton, Dickinson and Company, USA) plates, and cultured in 500 mL of tryptic soy broth medium (Becton, Dickinson and Company, USA). Then, these two bacteria were incubated at 28 °C while shaking (150 rpm) in a shaking incubator (HZ-9210K, Jiangsu, China) for 8 h. Species identity of the bacterial strains was confirmed by polymerase chain reaction (PCR) amplification of the 16S ribosomal RNA gene region with universal primer for bacteria, 27F/1492R [11]. Amplicons of approximately 1500 bp were obtained from PCR products and cloned into the pMD18-T vector for Sanger sequencing (Takara, Dalian, China). BLASTn analysis revealed that the amplicons from two strains shared over 99% nucleotide sequence identity with *A*. *hydrophila* and *A*. *veronii*, respectively.

At an OD 600 nm of 0.6, formalin was added to the bacterial solutions at a final concentration of 0.5%, and then bacterial solutions were inactivated at 28 °C for 24 h, during which the shake was taken three times. The bacterial cells were harvested by centrifugation (2683*g* at 4 °C for 5 min) from the above solutions, washed three times, and resuspended in phosphate buffer saline (PBS, 0.01 M, pH 7.4). Then, the bacterial cell suspensions were adjusted to a concentration of 1.0 × 10^10^ CFU/mL by a turbidimetric method with sterile PBS. Next, 10 μL bacterial cell suspension was spread onto the TSA plates with three duplicates and then incubated at 28 °C for 48 h for the detection of viable bacterial cells. The two kinds of bacterial cell suspensions were mixed in equal volumes, and the concentration of each bacterial cell in the mixture was 5.0 × 10^9^ CFU/mL, namely, inactivated bivalent *A*. *hydrophila*/*A*. *veronii* vaccine. The prepared vaccine was stored at 4 °C and used immediately the next day.

### Experimental design and protocol

This experiment was carried out on a commercial fish farm located in Yingshan County, Huanggang City, Hubei Province, China. Eight suspended net cages were set up in an industrial cement pond (length 8 m × width 5 m × height 2.5 m) equipped with the flow-through aquaculture system. The rectangular cages measured 1.2 m (length) × 1 m (width) × 2 m (height) and were made of blue polyethylene netting of 1 mm mesh size. The cages were tied to stakes and suspended about 0.4 m from the bottom of the pond. The cages were completely covered with nets to prevent predators and fish escape. The experimental water was derived from a local warm spring, and the water quality was fresh and pollution free. After purification by an aeration tank, the water quality parameters were within the normal range of tilapia farming. The pond was filled with purified water to a depth of 2 m, and the purified water was continuously poured into the pond with a flow rate of about 0.1 m^3^ s^−1^. The depth of water in the cage was kept at 1.6 m, resulting in a useful volume of 1.92 m^3^ in each cage. Feed residues and fish feces were removed from the sewage system at the bottom of the pond. Air was continuously supplied from pipes with holes in the bottom of the pond through an air blower.

Adult tilapias (NEW GIFT strain of *O*. *niloticus*) were reared in the above pond and domesticated for 2 weeks. All tilapias used for the experiment were sourced from the same batch of artificially incubated seeds. The fish were disinfected with 5 ppm KMnO_4_ for 30 s before acclimatization. Eight cages were divided into two groups (control group and vaccinated group) with quadruplicate cages per group. Following acclimation, 160 fish of nearly the same size were randomly divided into these cages (20 fish per cage). Fish in the vaccinated group (V) were intraperitoneally injected with 0.5 mL of the prepared vaccine, whereas fish in the control group (P) were injected with 0.5 mL of PBS (0.01 M, pH 7.4).

The cage experiment began on October 20, 2016, and lasted for 45 days. During the experiment period, the fish were fed with the commercial diet at a daily rate of 3% body weight, with floating feed pellets produced in Huai’an TianShen Feed Co., Ltd., China (crude protein ≥ 30.0%, crude fat ≥ 4.5%, crude fiber ≥ 9.0%, crude ash ≥ 12.0%, total phosphorus ≥ 0.8%, sodium chloride 0.4%–4.0%, lysine ≥ 1.7%, and moisture ≤ 12.5%). No abnormal behavior or mortality occurred throughout the experimental period. During the experimental procedure, the environmental factors remained stable, including no usage of fishery drugs, no large fluctuations, and consistent management. According to the average weights recorded weekly, the daily amount of feed for each cage was readjusted accordingly, and the water level was raised accordingly to maintain the stocking density. The water physicochemical parameters, including ammonia nitrogen (< 0.3 mg/L), dissolved oxygen (> 3 mg/L), water temperature (28 ± 2 °C), and pH (7–8.5) were maintained at an optimum level.

### Sample collection and processing

After acclimation, 12 tilapias were randomly selected and weighted as the initial body weight (0.24 ± 0.02 kg; Additional file 1. Supplementary Table 1). At the end of the experiment, tilapias of similar size were randomly sampled from all cages (three fish per cage) at 6 h after the last feeding. After collection, the tilapias were lightly anesthetized in the low dose of ethyl 3-aminobenzoate methanesulfonate (MS-222; Sigma, Germany) solution and immediately weighed. The weight gain (%) was calculated according to the following formulae: WG (%) = 100 × (FBW − IBW)/IBW; FBW is the final body weight, and IBW is the initial body weight. Then, blood samples were immediately obtained from the caudal vein of these fish with 5-mL syringes. The collected blood samples were divided into two fractions: One aliquot was allowed to clot at 4 °C for 4 h, and the serum was obtained after centrifugation at 2000*g* for 5 min at 4 °C. The collected serum was kept at −20 °C for subsequent analysis of blood biochemical parameters. Another aliquot was placed in a blood collection tube containing ethylenediaminetetraacetic acid dipotassium (EDTAK_2_) used for the subsequent analysis of hematological parameters. Then, samples of gill filaments (G), stomach contents (S), stomach mucosae (W), intestinal contents (C), and intestinal mucosae (M) were collected and processed according to the previous method [36]. The collected samples were placed in 1.5-mL centrifuge tubes and plunged into liquid nitrogen flash-freezing. Then, all samples were stored at −80 °C.

### Determination of hematological and biochemical parameters

After collection, the hematological parameters and serum biochemical parameters of the blood samples were determined immediately. An automated hematology analyzer (Sysmex XN9000-A, Kobe, Japan) was applied to detect the main hematological parameters, including red blood cells (RBC, 10^12^ L^−1^), white blood cells (WBC, 10^9^ L^−1^), hemoglobin (Hb, g L^−1^), packed-cell volume (PCV, %; also called hematocrit, HCT), mean corpuscular volume (MCV, fL), mean corpuscular hemoglobin (MCH, pg), mean corpuscular hemoglobin concentration (MCHC, g L^−1^), red blood cell distribution width (RDW, %), platelet (PLT, 10^9^ L^−1^), mean platelet volume (MPV, fL), and platelet distribution width (PDW, %). A fully automatic biochemical analyzer (Beckman Coulter AU5400, Pasadena, USA) was applied to test the serum biochemical parameters, including total protein (TP, g L^−1^), albumin (ALB, g L^−1^), globulin (GLO, g L^−1^), and albumin/globulin ratio (A/G). The contents of serum lysozyme (LZM; μg mL^−1^) and superoxide dismutase (SOD; U mL^−1^) were determined with commercial test kits (Nanjing Jiancheng Bioengineering Institute, China) according to the manufacturer’s instructions. Moreover, the serum antibody titers against *A*. *hydrophila* (anti-*A*. *hydrophila* titer) and *A*. *veronii* (anti-*A*. *veronii* titer) were determined with the agglutination effect of serum on antigens.

### DNA extraction, PCR amplification, and Miseq sequencing

All collected samples were prepared for genomic DNA extraction using QIAamp® DNA Stool Mini Kit (Qiagen, Germany) according to the manufacturer’s instructions. For each sample, duplicate DNAs were extracted and pooled together to reduce sampling and extraction bias. The concentration and quality of DNA were determined using a Nanodrop 2000 Spectrophotometer (Thermo Scientific, Waltham, MA, USA). Then, extracted DNA was diluted to 10 ng/μL for downstream research.

The universal primers 515F (5′-GTGCCAGCMGCCGCGGTAA-3′) and 909R (5′-CCCCGYCAATTCMTTTRAGT-3′) with 12 nt unique barcodes at the 5′-end of 515F were used to amplify the V4–V5 hypervariable region of 16S rRNA gene [37]. As described previously [36], the PCR reaction mixture (25 μL) consisted of 10 ng of DNA temple, 1 × PCR buffer, 1.5 mM MgCl_2_, each deoxynucleoside triphosphate at 0.4 μM, each primer at 1.0 μM, and 0.5 U of Ex Taq (TaKaRa, Dalian, China). PCR was conducted using the following program: initial denaturation at 94 °C for 3 min, followed by 30 cycles of denaturation at 94 °C for 40 s, annealing at 56 °C for 60 s, and elongation at 72 °C for 60 s, and a final extension at 72 °C for 10 min. Replicate PCR reactions were conducted for each sample, and the DNA library was constructed as described in the Illumina library preparation protocols. Finally, the purified DNA library was applied to an Illumina Miseq system for sequencing with the Reagent Kit v2 2×250 bp at the Environmental Genome Platform of Chengdu Institute of Biology [36].

### Extraction, detection, and annotation of intestinal metabolites

Metabolite extraction, detection, and annotation from the intestinal contents were conducted according to the previous method [36]. Briefly, 100 mg of fresh intestinal contents was subjected to a series of pretreatments for GC-MS detection, and then samples were determined for metabolites using an Agilent 7890A GC system coupled to an Agilent 5975C inert XL EI/CI mass spectrometric detector system (Agilent Technologies, Santa Clara, CA, USA) [36]. GC was performed on an HP-5MS capillary column (5% phenyl/95% methylpolysiloxane 30 m × 250 μm i.d., 0.25 μm film thickness, Agilent Scientific, Folsom, CA, USA) to separate the derivatives at a constant flow of 1 mL/min helium, and MS was determined by the full-scan method within the range of 35 to 750 (m/z). Using the XCMS (www.bioconductor.org) package in R software (v3.1.3), raw GC-MS data were processed to obtain the data matrices, including mass-to-charge ratio (m/z), retention time, and intensity. By using the Automatic Mass Spectral Deconvolution and Identification System, the annotation of metabolites was searched against commercially available databases, such as the National Institute of Standards and Technology and Wiley Registry Metabolomics Database. Then, the substances were qualitatively characterized by the alkane retention indices provided by the Golm Metabolome Database (http://gmd.mpimp-golm.mpg.de/), most of which were further confirmed by the standards. Finally, the data were normalized to the internal standard for further analyses. In summary, the experimental manipulations and analytical methods of intestinal metabolome are provided in detail (Additional file 2. Supplementary Methods).

### Bioinformatics and statistical analyses

According to the previous method [36], the paired-end reads from the raw DNA fragments were merged by FLASH software and quality-filtered by Trimmomatic with the following criteria. (1) The 300 bp reads were truncated at any site receiving an average quality score < 20 over a 50-bp sliding window, and then, the reads shorter than 50 bp or containing N-bases were removed. (2) The pair-end reads were merged into a sequence according to their overlap relationship, and the minimum overlap length was 10 bp. (3) The maximum mismatch ratio allowed in the overlap area of the merged sequence was 0.2. (4) The sequences were distinguished and corrected according to the barcodes and primer sequences. The number of mismatches allowed in the barcode was 0, and the maximum number of mismatched primers was 2. Operational taxonomic units (OTUs) were clustered with the 97% similarity cutoff using UPARSE (version 7.0 http://drive5.com/uparse/), and chimeric sequences were identified and removed using UCHIME. The taxonomy of each OTU representative sequence was analyzed by RDP Classifier (http://rdp.cme.msu.edu/) against the Bacterial Silva 16S rRNA database (SILVA SSU 138) using a confidence threshold of 0.8. Considering that the minimum number of effective sequences in stomach samples was significantly lower than that of other samples (Additional file 1: Supplementary Table 2), the stomach samples were separated from other samples for random resampling to prevent affecting the number of random resampled reads in other samples. The stomach content and mucosa samples were randomly resampled to a minimum read number of 2671, whereas the gill mucosa, intestinal content, and mucosa samples were randomly resampled to a minimum read number of 5810.

Alpha diversity was estimated with the richness indices of the observed richness (OTUs) and Chao, the diversity indices of Shannon and Simpson, and Good’s coverage (coverage). Beta diversity was analyzed with principal coordinate analysis (PCoA) and Analysis of similarity (ANOSIM) based on the Bray–Curtis metric. The linear discriminant analysis (LDA) effect size (LEfSe) is an algorithm used for the high-dimensional biomarker discovery and explanation that identify genomic features characterizing the differences under two or more biological conditions [38]. In this paper, LEfSe analysis was performed to identify microbial biomarkers and functional differences with an alpha parameter of 0.05 and an LDA threshold value of 2.0. Differences between the two independent groups were evaluated using Welch’s *t*-test (Past, version 3.15). The *p*-value was corrected using the Bonferroni method. The functional profiles of the bacterial communities were predicted using the Tax4Fun from the KEGG pathways [39]. The statistical analysis of the microbial function was performed using the Statistical Analysis of Metagenomics Profiles [40]. Data were expressed as mean ± standard deviation (mean ± SD, *n* = 4), and the significance level of the difference was set at 0.05, 0.01, or 0.001.

## Results

### Effect of vaccination on growth performance and survival rate

At the beginning of the experiment, the average weight of tilapia in the initial group was 0.24 ± 0.02 kg (Additional file 1: Supplementary Table 1). At the end of the experiment, the average weight of tilapia in the control group was 0.32 ± 0.04 kg, whereas that in the vaccinated group was 0.33 ± 0.04 kg (Additional file 1: Supplementary Table 1). No significant difference in weight gain of tilapia was found between the control and vaccinated groups (Welch *t*-test, *p* > 0.05). In addition, no death of tilapia was observed in all net cages during the experiment, that was, the survival rate was 100%. These results indicated that inactivated vaccination had no negative effect on the growth performance and survival rate of adult tilapia.

### Effect of vaccination on hematological parameters and serum biochemical indices

**Fig. 1.**
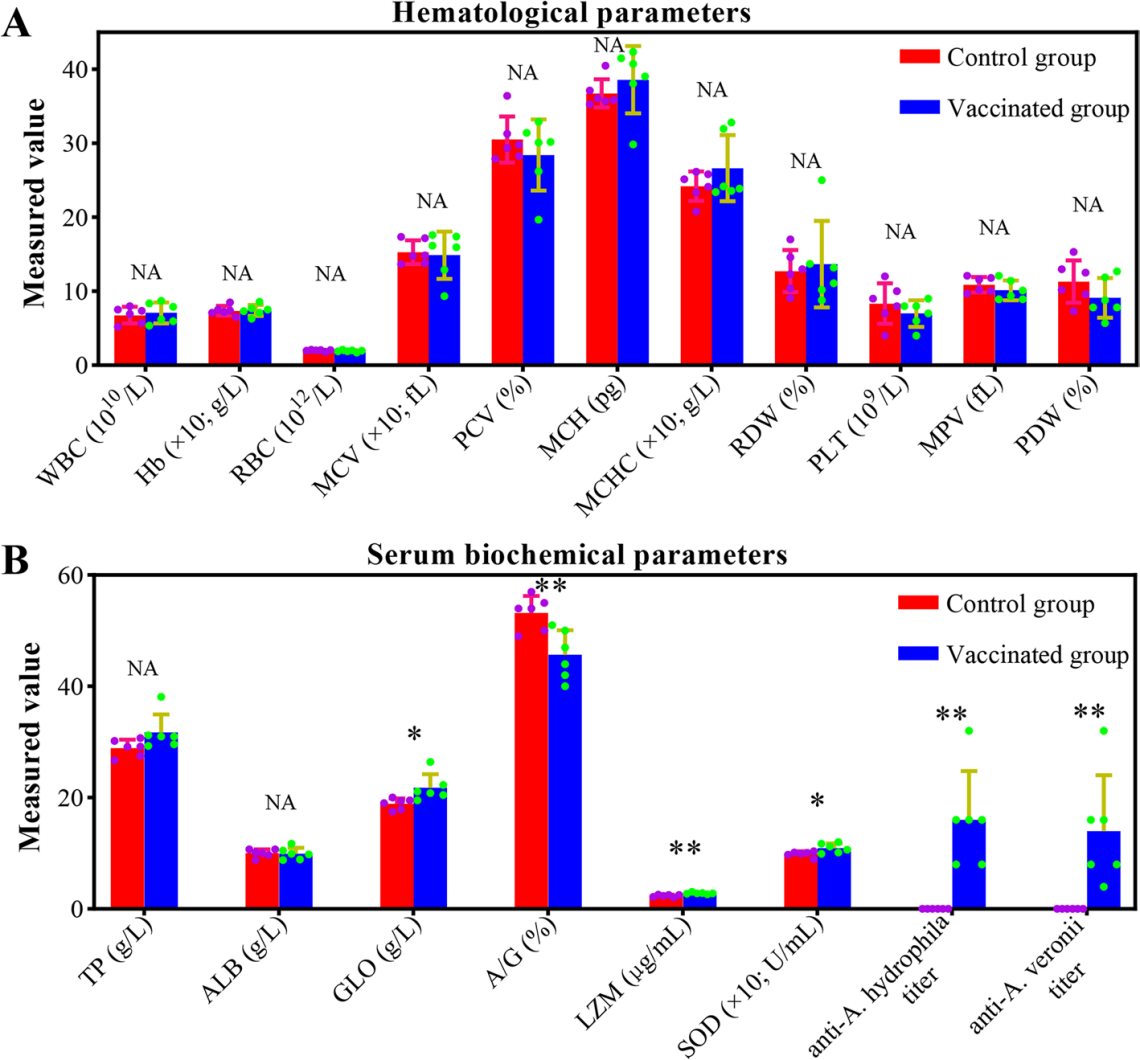
**A** Effect of vaccination on hematological parameters of tilapia. **B** Effect of vaccination on serum biochemical parameters of tilapia. ** means extremely significant differences, that is *p* < 0.01; * means significant differences, that is *p* < 0.05; NA means no significant differences, that is *p* > 0.05

No significant differences (Welch *t*-test, *p* > 0.05) were found in all hematological parameters of tilapia between the control and vaccinated groups (Fig. 1A). Moreover, serum total protein and albumin levels showed no significant differences (Welch *t*-test, *p* > 0.05) between the control and vaccinated groups (Fig. 1B). The above results suggested that the health and nutritional status of tilapia were not affected by vaccination. Conversely, the serum globulin, albumin/globulin ratio, lysozyme content, and superoxide dismutase activity of tilapia in the vaccinated group were significantly higher (Welch *t*-test, *p* < 0.05 or *p* < 0.01) than those in the control group (Fig. 1B), indicating that the nonspecific immunity of tilapia was enhanced after vaccination. In addition, serum antibody titers against *A*. *hydrophila* and *A*. *veronii* were 16 ± 8.76 and 14 ± 10.04 in the vaccinated group, respectively, whereas no serum antibody titer was detected in the control group (Fig. 1B), suggesting that vaccination induced the production of specific antibodies in tilapia.

### Effect of vaccination on the diversity, structure, and distribution of symbiotic microbiota

**Fig. 2.**
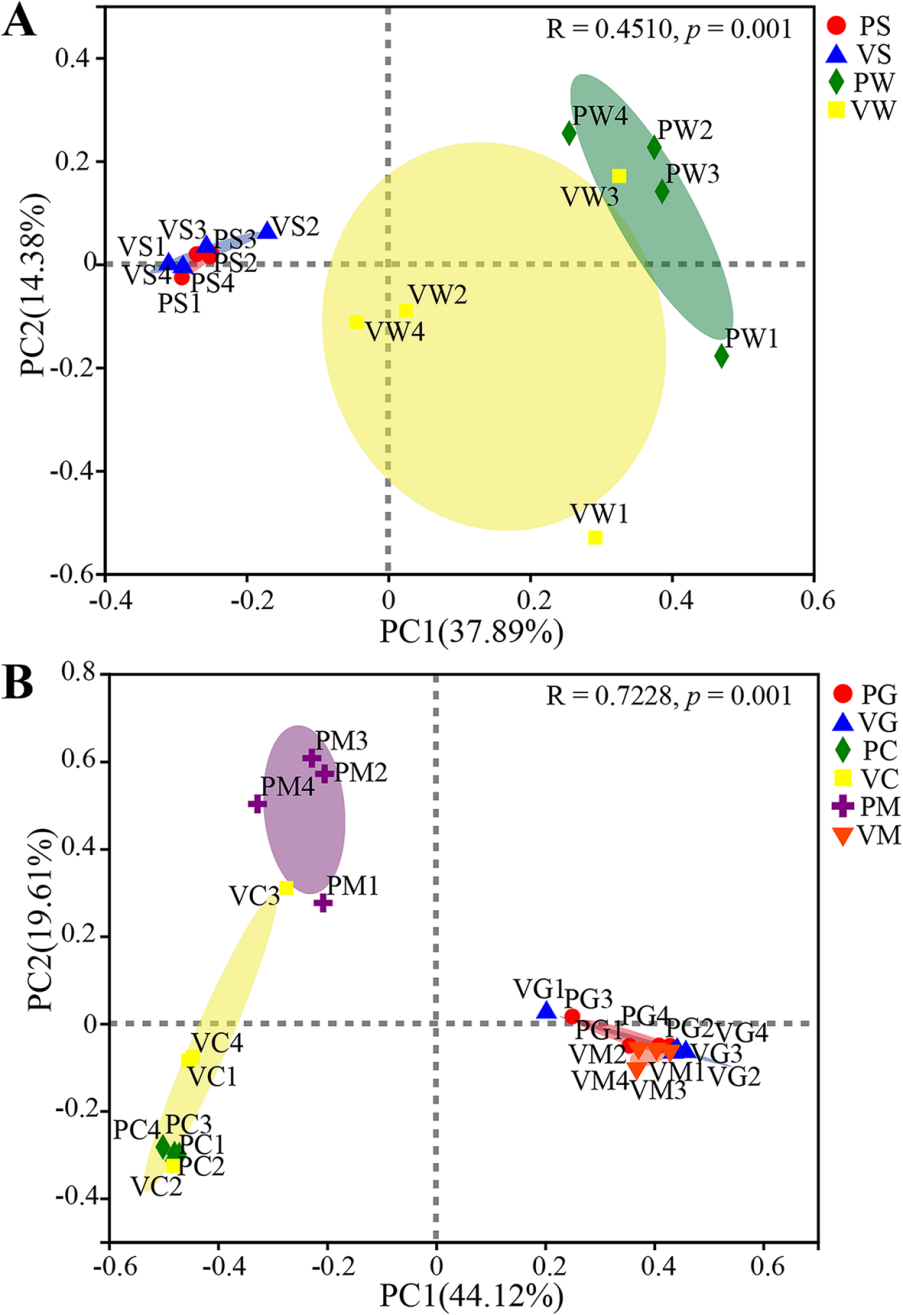
Principal coordinate analysis based on the Bray–Curtis metric of the bacterial communities: **A** structural analysis of the bacterial communities in the stomach content and mucosa samples, **B** structural analysis of the bacterial communities in the gill and intestinal samples. PS1-PS4: stomach contents in the control group; VS1-VS4: stomach contents in the vaccinated group; PW1-PW4: stomach mucosae in the control group; VW1-VW4: stomach mucosae in the vaccinated group; PG1-PG4: gill mucosae in the control group; VG1-VG4: gill mucosae in the vaccinated group; PC1-PC4: intestinal contents in the control group; VC1-VC4: intestinal contents in the vaccinated group; PM1-PM4: intestinal mucosae in the control group; VM1-VM4: intestinal mucosae in the vaccinated group

After quality filtering, 480,387 valid reads (ranging from 2671 to 70,742 per sample) were generated from the 40 samples. After resampling, the Good’s coverage of all samples ranged from 97.53 to 99.97% (Additional file 1: Supplementary Table 3). The rarefaction curves plateaued in all samples (Additional file 1: Supplementary Figures 1A and D), and the Shannon curves were stable (Additional file 1: Supplementary Figures 1B and C). These results indicated that the majority of the microbial diversity present in the samples was detected. Statistical analysis showed that no significant differences (Welch *t*-test; *p* > 0.05) were found in the richness (OTUs and Chao) and diversity (Shannon and Simpson) of the symbiotic microbiotas between the control and vaccinated groups (Additional file 1: Supplementary Table 3), indicating that vaccination had no significant effect on the diversity and richness of the symbiotic microbiota. ANOSIM revealed that vaccination significantly (*R* = 0.9896, *p* = 0.034) affected the bacterial community structure of intestinal mucosa, whereas vaccination did not significantly (*p* > 0.05) affect the bacterial community structure of the other sample types (gill mucosa, stomach content and mucosa, and intestinal content) (Table 1). The PCoA plot visualized the ANOSIM results, showing distinct separations of the bacterial communities in the intestinal mucosa between the control and vaccinated groups but no significant separation of the bacterial communities in the other sample types between the control and vaccinated groups (Fig. 2A, B). Overall, the two principal coordinates obtained from PCoA explained 52.27% (Fig. 2A) and 63.73% of the variations among all samples (Fig. 2B).

**Table 1.** Analysis of similarity (ANOSIM) of the structure and function of the bacterial communities based on the Bray–Curtis metric

Group	Community structure		Community function	
	R	***p***	R	***p***
PS vs. VS	−0.1146	0.847	0.0104	0.333
PW vs. VW	0.2188	0.105	0.0313	0.298
PG vs. VG	−0.1667	0.982	−0.125	0.926
PC vs. VC	0.0625	0.227	0.1458	0.199
PM vs. VM	0.9896	0.034	0.9583	0.032

Permutation *N* = 999; R is assessed by permuting the grouping vector to obtain the empirical distribution of R under the null model; a *p*-value less than 0.05 means significant

At the phylum level, Bacteroidota, Proteobacteria, and Fusobacteriota were the dominant phyla in the stomach contents, whereas Proteobacteria, Firmicutes, and Bacteroidota were the dominant phyla in the stomach mucosae (Additional file 1: Supplementary Figure 2A). In the gill mucosae, the bacterial phyla were dominated by Proteobacteria (over 95%), followed by Actinobacteria and Firmicutes (Additional file 1: Supplementary Figure 2B). Regarding the intestinal samples, the intestinal contents were dominated by Fusobacteriota, followed by Firmicutes and Proteobacteria, whereas the intestinal mucosae were dominated by Proteobacteria, followed by Firmicutes, Fusobacteriota, Cyanobacteria, and Actinobacteria (Additional file 1: Supplementary Figure 2B). At the phylum level, compared with the control group, only the relative abundance of Actinobacteriota in the intestinal mucosae was significantly increased (Welch *t*-test, corrected *p* < 0.01) in the vaccinated group, whereas no significant differences (Welch *t*-test, corrected *p* > 0.05) were found in other taxa in the vaccinated group (Additional file 1: Supplementary Figure 3). In addition, at the genus level, differences in the microbial distribution between the control and vaccinated groups were observed (Additional file 1: Supplementary Figures 4A and B).

### Effect of vaccination on the taxonomic composition of symbiotic microbiota

The differential OTUs of symbiotic microbiota at different sites between the control and vaccinated groups were further analyzed by LEfSe. By comparing the gill mucosae, the results revealed that *Limnobacter thiooxidans* OTU215 was significantly enriched in the vaccinated group, whereas *Roseomonas gilardii* OTU21, *Ochrobactrum* OTU37, and Chloroplast sp. OTU87 were significantly enriched in the control group (Additional file 1: Supplementary Figure 5A). LEfSe identified 15 discriminative features (LDA score > 2) in the stomach contents between the control and vaccinated groups, of which 5 OTUs were significantly enriched in the stomach contents of the vaccinated group, including *Macellibacteroides* OTU318, Alphaproteobacteria sp. OTU232, Bacteroidetes vadinHA17 sp. OTU236, Paludibacteraceae sp. OTU247, and unclassified bacteria sp. OTU49 (Additional file 1: Supplementary Figure 5B). Conversely, 10 additional OTUs were significantly enriched in the stomach contents of the control group (LDA score > 2), mainly including Chloroplast sp. OTU81, HOC36 sp. OTU163, Calditrichaceae sp. OTU112, Lacunisphaera sp. OTU1, Fibrobacteraceae sp. OTU43, *Sporobacter* OTU72, *Acidaminobacter* OTU371, and *Caulobacter* OTU151 (Additional file 1: Supplementary Figure 5B). Regarding the stomach mucosae, 13 OTUs were significantly enriched in the vaccinated group, namely, *L*. *thiooxidans* OTU421 and OTU395, Comamonadaceae sp. OTU373, Chloroplast sp. OTU81, *Armatimonas* OTU107, *Clostridium sensu stricto* 14 OTU379, Mitochondria sp. OTU52, *Polaromonas* OTU297, *Fibrobacter* OTU78, Actinomycetaceae sp. OTU393, *Flavobacterium* OTU378, Bacteroidetes BD2-2 sp. OTU245, and *Clostridium sensu stricto* 1 OTU315. Conversely, 10 OTUs were significantly enriched in the control group, namely, unclassified bacteria sp. OTU204, OTU177, and OTU173, *Pelomonas* OTU184, *Thauera* OTU190, *Arcobacter* OTU175, *Arenimonas* OTU228, *Parvibium* OTU65, *Haliangium* OTU217, and *Armatimonas* OTU278 (Additional file 1: Supplementary Figure 5C).

**Fig. 3.**
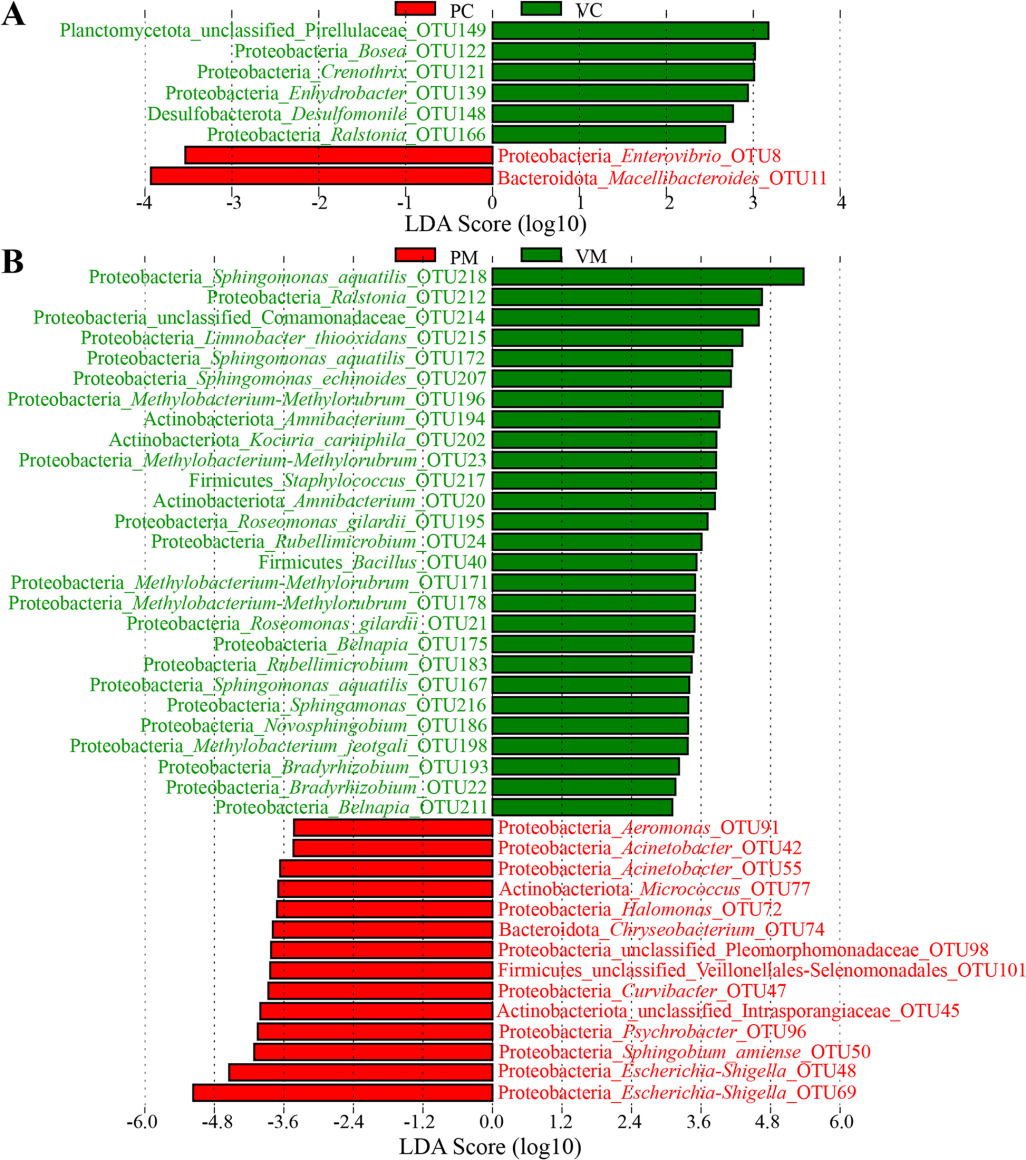
Comparison of the intestinal bacterial communities at the OTU level by LEfSe: **A** comparison of the bacterial communities in the intestinal contents between the control and vaccinated groups, **B** comparison of the bacterial communities in the intestinal mucosae between the control and vaccinated groups. The highlighted taxa are enriched in the group that corresponds to each color; LDA scores can be interpreted as the degree of difference in the relative abundance of OTUs; PC: intestinal contents in the control group, VC: intestinal contents in the vaccinated group, PM: intestinal mucosae in the control group, VM: intestinal mucosae in the vaccinated group

By comparing the intestinal contents, the results revealed that Pirellulaceae sp. OTU149, *Bosea* OTU122, *Crenothrix* OTU121, *Enhydrobacter* OTU139, *Desulfomonile* OTU148, and *Ralstonia* OTU166 were significantly enriched in the vaccinated group, whereas *Macellibacteroides* OTU11 and *Enterovibrio* OTU8 were significantly enriched in the control group (Fig. 3A). LEfSe identified 41 differential OTUs in the intestinal mucosae between the control and vaccinated groups (LDA > 2), of which 14 OTUs were significantly enriched in the control group, including *Escherichia*–*Shigella* OTU69 and OTU48, *Sphingobium amiense* OTU50, *Psychrobacter* OTU96, Intrasporangiaceae sp. OTU45, *Curvibacter* OTU47, Veillonellales–Selenomonadales sp. OTU101, Pleomorphomonadaceae sp. OTU98, *Chryseobacterium* OTU74, *Halomonas* OTU72, *Micrococcus* OTU77, *Acinetobacter* OTU55 and OTU42, and *Aeromonas* OTU91 (Fig. 3B). Conversely, 27 other OTUs were significantly enriched in the vaccinated group (LDA score > 2), mainly including *Sphingomonas aquatilis* OTU218, OTU172, and OTU167; *Sphingomonas echinoides* OTU207; *Sphingomonas* OTU216; *Ralstonia* OTU212; Comamonadaceae sp. OTU214; *L*. *thiooxidans* OTU215; *Methylobacterium*–*Methylorubrum* OTU196, OTU23, OTU171, and OTU178; *Methylobacterium jeotgali* OTU198; *Amnibacterium* OTU194 and OTU20; *Kocuria carniphila* OTU202; *Staphylococcus* OTU217; *R*. *gilardii* OTU195 and OTU21; *Rubellimicrobium* OTU24 and OTU183; *Bacillus* OTU40; *Belnapia* OTU175 and OTU211; *Novosphingobium* OTU186; and *Bradyrhizobium* OTU193 and OTU22 (Fig. 3B).

### Effect of vaccination on the composition and structure of intestinal metabolites

**Fig. 4.**
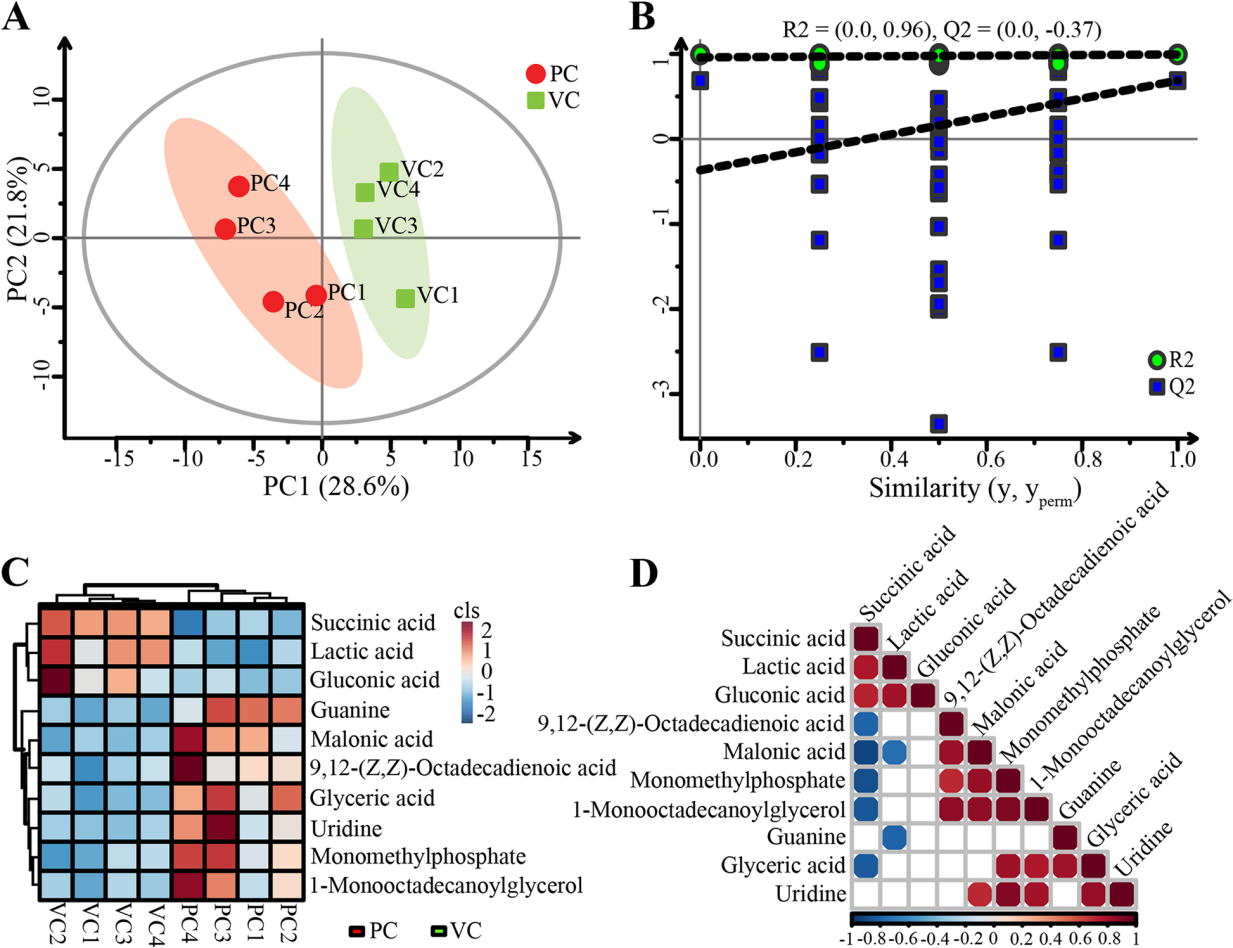
Differential analysis of intestinal metabolites between the control and vaccinated groups: **A** partial least squares-discriminant analysis (PLS-DA), **B** permutations plot of metabolome data processing, **C** clustering heatmap analysis of differential metabolites, **D** the heatmap showing the association of differential metabolites. The correlation coefficient is represented by different colors: orange-red represents positive correlation (*p* < 0.05), blue-green represents negative correlation (*p* < 0.05). The larger circle represents the greater correlation coefficient

GC-MS metabolomics was used to assess the response of intestinal metabolites to vaccination. First, the quality assurance for the original data was conducted, and the 30% characteristic peak ratio reached 98.9%, indicating that the data quality was acceptable (Additional file 1: Supplementary Figure 6A). Then, the quality control (QC) for original data was performed, and QC samples were concentrated in the PCA diagram, indicating that the data were stable and reliable (Additional file 1: Supplementary Figure 6B). To distinguish differences between groups, partial least squares-discriminant analysis (PLS-DA) was further used to establish the relationship model between metabolite level and grouping. The explainable rates of model X variable and model Y variable were 0.577 and 0.994, respectively, and the number of principal components for automatic fitting was 3. The PLS-DA score plot showed a significant separation of tilapia intestinal metabolites between the control and vaccinated groups (Fig. 4A). Principal component PC1 accounted for 28.6% of the variables, and PC2 accounted for 21.8% of the variables. Overall, the two principal components of PLS-DA accounted for 50.4% of the variables in all samples (Fig. 4A). The permutation plot showed that the intersection of the regression line at Q2 was less than 0, so the current model was not over-fitted, and the above statistical results were valid (Fig. 4B).

In this paper, 95 different metabolites were detected in intestinal contents, including amino acids, lipids, carbohydrates, nucleotides, and vitamins. By comparing intestinal metabolites between the control and vaccinated groups, 10 differential metabolites were detected, which were mainly related to carbohydrate metabolism, lipid metabolism, and nucleic acid metabolism (Fig. 4C). Compared with the control group, the concentrations of lactic acid, succinic acid, and gluconic acid were significantly higher (*p* < 0.05) in the vaccinated group, whereas the concentrations of the seven other intestinal metabolites were significantly lower (*p* < 0.05) in the vaccinated group, including monomethylphosphate, 9,12-(Z,Z)-octadecadienoic acid, 1-monooctadecanoylglycerol, glyceric acid, malonic acid, uridine, and guanine (Fig. 4C). The correlation analysis of metabolites showed the three metabolites with significantly higher concentrations in the vaccinated group (*p* < 0.05) were significantly positively correlated (*p* < 0.05), and most of the seven metabolites with significantly lower concentrations in the vaccinated group were also significantly positively correlated (*p* < 0.05). Moreover, succinic acid was significantly negatively correlated (*p* < 0.05) with four lipid metabolites, namely, monomethylphosphate, 9,12-(Z,Z)-octadecadienoic acid, 1-monooctadecanoylglycerol, and glyceric acid (Fig. 4D).

### Correlation between differential intestinal metabolites and intestinal microorganisms

**Fig. 5.**
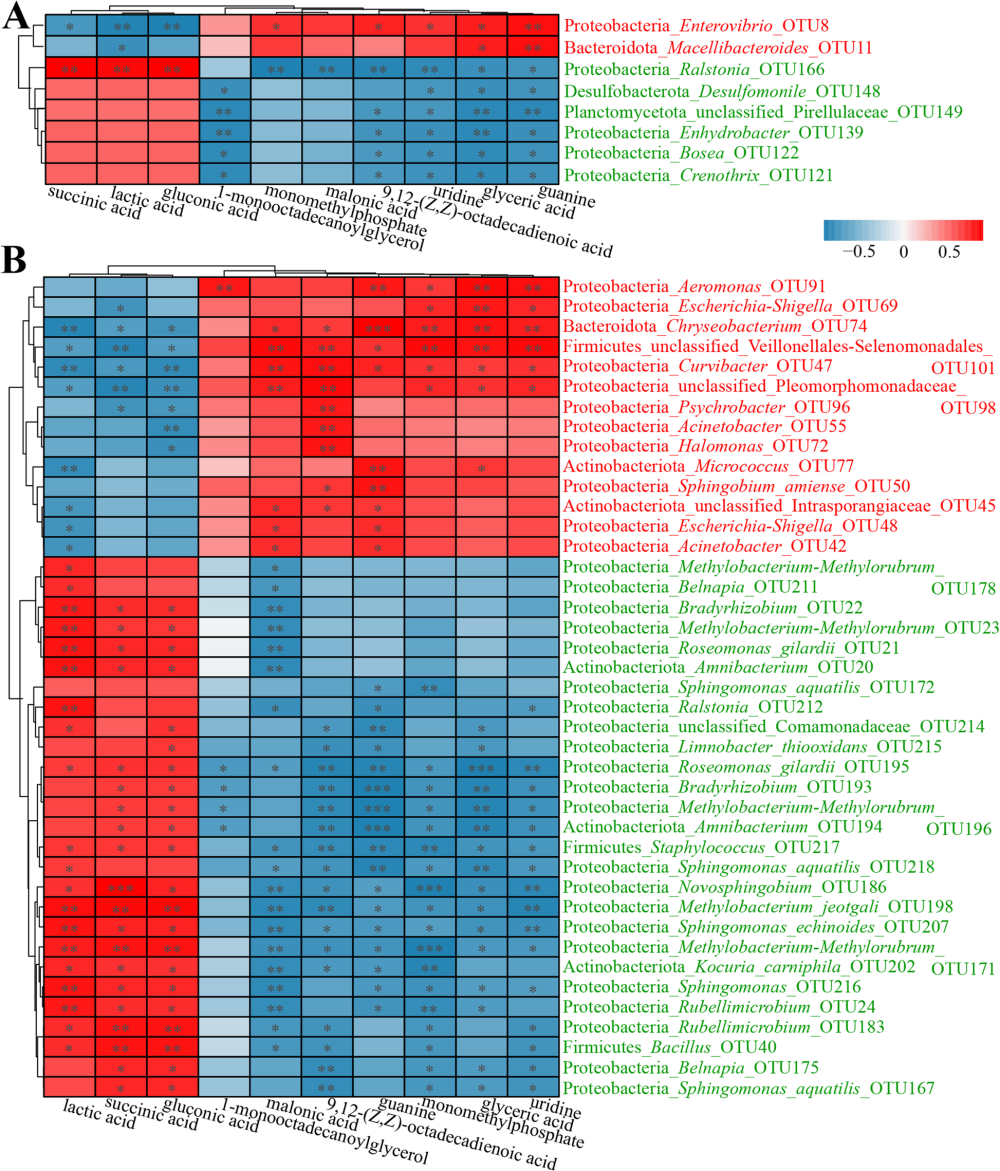
Correlation analyses between the differential intestinal microbes and metabolites: **A** correlation analysis between the differential metabolites and the differential microbes in the intestinal contents, **B** correlation analysis between the differential metabolites and the differential microbes in the intestinal mucosae. The correlation coefficient is represented by different colors: red represents positive correlation, blue represents negative correlation. *Represents significantly negative or positive correlations: * *p* < 0.05; ** *p* < 0.01; *** *p* < 0.001

Further correlation analyses were conducted between differential intestinal OTUs and 10 differential intestinal metabolites (Fig. 5A, B). Compared with the control group, the intestinal microorganisms that decreased significantly in the vaccinated group were positively correlated with changes in seven metabolites that reduced significantly in the vaccinated group and were significantly (*p* < 0.05) positively correlated with some of these seven metabolites. Conversely, these microorganisms were negatively correlated with the changes in three metabolites that increased significantly in the vaccinated group and were significantly (*p* < 0.05) negatively correlated with some of these three metabolites (Fig. 5A, B). Compared with the control group, the intestinal microorganisms that increased significantly in the vaccinated group were negatively correlated with changes in seven metabolites that decreased significantly in the vaccinated group and were significantly (*p* < 0.05) negatively correlated with most of these metabolites. Conversely, these microorganisms were positively correlated with changes in three metabolites that increased significantly in the vaccinated group, especially *Ralstonia* OTU166 in the intestinal contents, and most of the differential microorganisms in the intestinal mucosae were significantly (*p* < 0.05 or 0.01) positively correlated with these three metabolites (Fig. 5A, B). These results indicated that the influence of vaccination on intestinal metabolism was closely related to the alterations of intestinal microbes.

### Effect of vaccination on the predictive function of symbiotic microbiota

**Fig. 6.**
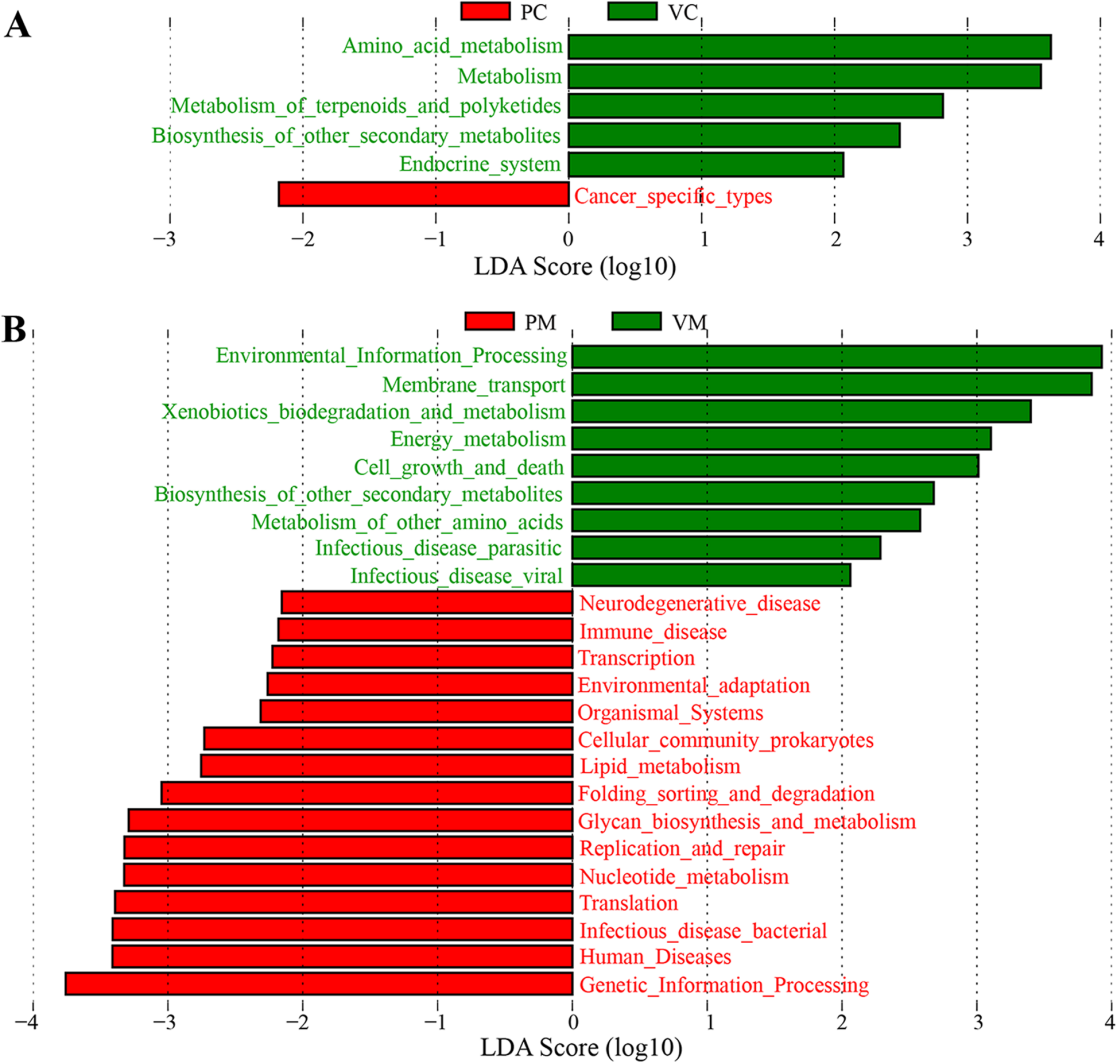
Comparison of functional composition of bacterial communities by Tax4Fun: **A** comparison of functional composition of bacterial communities in the intestinal contents between the control and vaccinated groups, **B** comparison of functional composition of bacterial communities in the intestinal mucosae between the control and vaccinated groups. PC: intestinal contents in the control group; VC: intestinal contents in the vaccinated group; PM: intestinal mucosae in the control group; VM: intestinal mucosae in the vaccinated group

Tax4Fun was used to predict functional profiles of tilapia symbiotic microbiota. ANOSIM showed that vaccination had no significant (*p* > 0.05) effect on the functional structure of bacterial communities in gill mucosae, stomach mucosae, stomach contents, and intestinal contents but had a significant (*p* < 0.05) effect on the functional structure of bacterial communities in intestinal mucosae (Table 1). The PCA plot visualized the ANOSIM results, showing the significant separations of the functional profile in the intestinal mucosae between the control and vaccinated groups (Additional file 1: Supplementary Figure 7). Compared with the control group, the functional pathways related to metabolism, especially amino acid metabolism, metabolism of terpenoids and polyketides, and biosynthesis of other secondary metabolites were significantly enriched (LDA > 2.0) in the intestinal digesta-associated microbiota of the vaccinated group (Fig. 6A). In addition, the functional pathways related to xenobiotic biodegradation and metabolism, energy metabolism, biosynthesis of other secondary metabolites, and metabolism of other amino acids were significantly enriched (LDA > 2.0) in the intestinal mucosa-associated microbiota of the vaccinated group. Conversely, the functional pathways involved in bacterial infectious disease, nucleotide metabolism, glycan biosynthesis and metabolism, lipid metabolism, and immune disease were significantly enriched (LDA > 2.0) in the intestinal mucosa-associated microbiota of the control group (Fig. 6B).

## Discussion

Although vaccines used to prevent bacterial diseases have great potential in aquaculture, their development remains extremely complex [4]. To date, the intestinal microbiota of tilapia has been widely studied, but the evidence for the intestinal microbiota alteration of tilapia after vaccine administration is limited. This paper evaluated the alterations of symbiotic microbiota in tilapia after inactivated vaccination and its correlation with immune function and metabolic status due to the important role of complex symbiotic microbiota in host immunity and metabolism [17]. Intestinal microorganisms are closely related to the induction of mucosal immunity [23]. Moreover, fish mucous epithelial cells can secrete immune factors that interact with the mucosal microbiota, thus shaping the colonization of symbiotic microbiota [24]. Considering the vaccine-mediated protective immunity, evaluating the response of fish symbiotic microbiome especially the mucosal microbiome to vaccination will contribute to elucidating the interaction mechanism between the vaccine and fish symbiotic microbiome.

The hematological parameters and biochemical indices can provide information about the host’s immunity and metabolism to a certain extent and are considered valuable biological indicators to assess the health status and physiological condition of fish [41]. This paper found that vaccination had no significant effect on the hematological parameters, indicating that inactivated vaccination did not affect the health status and physiological condition of fish. Moreover, the present results showed that vaccination had no significant effect on body weight gain and survival rate of tilapia, thus supporting the above view. However, the serum globulin, albumin/globulin ratio, lysozyme content, superoxide dismutase activity, and antibody titers against *A*. *hydrophila* and *A*. *veronii* increased significantly after vaccination, indicating that vaccination enhanced the nonspecific and specific immune functions of tilapia, which was consistent with previous studies [42–45]. The enhancement of immune function indicated that inactivated vaccine inoculated in this paper had a protective immune effect on tilapia, which was also the premise for the further evaluation of other effects of the vaccine [5].

Fish gills and gastrointestinal mucosae are the main routes of pathogen invasion, whose mucosal surfaces contain a large number of symbiotic microbes as biological barriers [23]. Previous studies on tilapia demonstrated that the diversity, structure, and composition of symbiotic microbiota were significantly different across gastrointestinal regions and sample types [36, 46]. Therefore, distinguishing tilapia resident and transient microorganisms is vital for investigating microbial function [24]. Recently, the composition of intestinal microbiota has been considered the major factor affecting vaccine efficacy in humans [47]. Certain members of the symbiotic microbiota are closely related to the production of mucosal immunity [23, 24]. Several reports showed that the specific microbial composition of the host was associated with differential vaccine responses [48, 49]. Microbial diversity was used to assess whether vaccination disrupted the symbiotic microbiota. The current results showed that vaccination had no significant impact on the richness and diversity indices of tilapia symbiotic microbiota, that is, vaccination did not destabilize the symbiotic microbiota, which was consistent with previous viewpoints [33, 35]. However, vaccination significantly changed the structure of intestinal mucosa-associated microbiota but did not significantly change the microbial structure of four other sites. Vaccination had region-specific effects on the symbiotic microbiota of tilapia and the most evident effect on intestinal mucosa-associated microbiota. Given the vaccine-mediated protective mucosal immunity [4], the inactivated vaccine might have a significant effect on intestinal mucosal immunity but a relatively light effect on gill and stomach mucosal immunity. Therefore, further studies should focus on the interaction mechanism between intestinal mucosal immunity and intestinal mucosa-associated microbiota. Previous studies have demonstrated that vaccination induces systemic and mucosal immune responses in fish [33, 50], and fish immune function plays an important role in maintaining mucosal microbial homeostasis [24, 25]. Thus, inactivated vaccination in this study may affect fish mucosal microbiota by enhancing immune function [51].

A previous study showed that a new recombinant *A*. *hydrophila* vaccine could effectively induce tissue immune response and significantly reduce the relative abundance of *Aeromonas* in the intestinal contents of grass carp [33]. However, the present results showed the relative abundance of Aeromonas did not significantly decrease in the intestinal contents of tilapia. This inconsistency might be related to differences in the type and dose of the vaccine and the type of vaccinated host [4]. The relative abundance of *Aeromonas* in the intestinal mucosae significantly decreased after vaccination in this paper, which might be due to the production of specific antibodies induced by the vaccine. Moreover, the relative abundance values of *Macellibacteroides* and *Enterovibrio* in the intestinal contents and *Escherichia*–*Shigella*, *Acinetobacter*, *Micrococcus*, and *Chryseobacterium* in the intestinal mucosae decreased significantly after vaccination. *Enterovibrio* promotes the production of indole, a toxin harmful to intestinal lactic acid bacteria in excess [52, 53]. The bacterial species within *Escherichia*–*Shigella* and *Acinetobacter* are common potential opportunistic pathogens of fish [54]. *Micrococcus* is generally considered a saprophytic or symbiotic microorganism and may be an opportunistic pathogen, especially in hosts with a compromised immune system, for example, coinfection and repeated infection of *Micrococcus lysodeikticus* and WSSV could cause severe infectious disease in shrimp [55]. *Chryseobacterium* can cause exogenous infection and endogenous infection due to the low immunity of the host and the unreasonable use of antibiotics [56]. As mentioned above, inactivated *Aeromonas* vaccination might have potential protective effects on tilapia intestinal infection against *Aeromonas* and other potential pathogens, which was consistent with a significant reduction in the abundance of pathways related to bacterial infectious disease and immune disease in the predictive function of intestinal mucosa-associated microbiota after vaccination (Fig. 6B). Mucosal vaccines are widely believed to be an attractive strategy for the control of emerging infectious diseases because they can effectively induce antigen-specific and systemic mucosal immune responses, and reduce the colonization of mucosal pathogens [57]. Therefore, the exploitation of attenuated strains of these mucosal pathogens will facilitate the development of fish mucosal vaccines in the aquaculture industry. In addition, the relative abundances of many intestinal microbes increased significantly after vaccination. Probiotics are widely believed to improve vaccine effectiveness by regulating the intestinal microbiota [31, 32] because the dysbiosis of intestinal microbiota can affect vaccine efficacy [29, 30]. Therefore, these intestinal symbiotic microbes that increased significantly after vaccination might serve as bacterial indicators for probiotic development to enhance vaccine efficacy against vaccinated fish.

The present results showed that after vaccination, the concentrations of carbohydrate-related metabolites significantly increased, but the concentrations of several lipid-related metabolites significantly decreased in the tilapia intestines (Fig. 4C), suggesting that vaccination might affect the carbohydrate and lipid metabolism in tilapia intestines. It was corroborated by the results of functional prediction, which showed that the abundance of the functional pathway for metabolism increased significantly in the intestinal digesta-associated microbiota after vaccination (Fig. 6A), and the abundance of lipid metabolism pathway reduced significantly in the intestinal mucosa-associated microbiota after vaccination (Fig. 6B). The immune system can affect the intestinal microbiota and participate in lipid metabolism in different forms [58]. On the contrary, intestinal microbiota can regulate intestinal enteroendocrine function and host metabolism through the immune pathway [59]. Thus, complex interactions exist among intestinal symbiotic microbiota, host immunity, and metabolism [60, 61]. Based on the direct stimulation effect of vaccination on fish immune function, vaccination might affect intestinal microbiota as well as intestinal metabolism by stimulating the fish immune system [33, 35]. The above statements suggested the presence of consistency between the composition or function of intestinal microbiota and intestinal metabolites [36]. Moreover, the abundance of xenobiotic biodegradation and metabolism pathway in intestinal mucosa-associated microbiota increased significantly after vaccination, suggesting that mucosa-associated microbiota might be more capable of degrading xenobiotics to protect mucosal tissues from exogenous damage after vaccination. Although some of the predicted differential metabolic pathways were not shown in the differential intestinal metabolites, the current results still suggested that changes in intestinal metabolites might be associated with functional changes in intestinal microbiota after vaccination [36]. Therefore, the intestinal metabolites that increased significantly after vaccination might serve as metabolic indicators for prebiotic development to enhance vaccine efficacy against vaccinated fish.

Further correlation analysis showed that after vaccination, a positive correlation between the intestinal differential microorganisms and metabolites with the same variation trend was observed, whereas a negative correlation between the intestinal differential microorganisms and metabolites with the opposite variation trend was noted (Fig. 5A, B). Significant or extremely significant correlations (*p* < 0.05 or 0.01) were found between most of the intestinal differential microorganisms and metabolites, for example, *Ralstonia* OTU166 in the intestinal contents was significantly (*p* < 0.01) positively correlated with almost all of the differential metabolites. In addition, the intestinal metabolites with the same change trend showed a certain degree of correlation (Fig. 4D). Each of the differential metabolites was positively correlated with the relative abundance values of some microorganisms and negatively correlated with the relative abundance values of other microorganisms (Fig. 5A, B). This phenomenon indicated that intestinal metabolites produced by the former may inhibit the latter, suggesting that intestinal metabolites might directly or indirectly drive and regulate population competition in the bacterial community [62]. The above results indicated that intestinal microbes might mediate the effect of vaccination on the intestinal metabolism of tilapia. Thus, in addition to evaluating the biological effects of vaccines from the perspective of immune protection, the symbiotic microbiota and metabolic function of the host may be potential indicators for evaluating vaccine efficiency. As a reference, although a safe, effective vaccine currently holds the greatest promise for controlling infectious diseases, hesitancy to accept vaccines remains common [63]. Therefore, relevant studies on the effect of vaccination on human symbiotic microbiota and metabolic function are urgently carried out.

## Conclusions

This paper for the first time revealed the response of tilapia symbiotic microbiota to vaccination and its correlation with host immunity and metabolism. The results showed that vaccination significantly affected the structure and composition of intestinal mucosal microbiota, and significantly reduced the relative abundance of potential opportunistic pathogens such as *Aeromonas* in intestinal mucosae. Combined with the enhancement of immune function after vaccination, inactivated bivalent *Aeromonas* vaccination might affect the intestinal symbiotic microbiota by stimulating immunity and thus have a potential protective effect against intestinal *Aeromonas* infection in tilapia. The findings revealed that vaccination significantly affected the carbohydrate and lipid metabolism in tilapia intestines, which was further verified by the predictive function of intestinal microbiota. Further correlation analyses revealed significant correlations between most of the intestinal differential microorganisms and metabolites, suggesting that the intestinal microbes might mediate the effect of vaccination on the intestinal metabolism of tilapia. The intestinal microbes and metabolites that increased significantly after vaccination might serve as valuable indicators for probiotics or prebiotics development to enhance vaccine efficacy against vaccinated fish. These observations provided a new perspective for understanding the mechanism of vaccine protection from the symbiotic microbiota and metabolic function. Given the current widespread use of human vaccines, our findings have implications for the potential influence of vaccination on human symbiotic microbiota and metabolism.

## Supplementary Information

**Additional file 1: Supplementary Table 1.** Results of tilapia body weight (kg) in different groups. **Supplementary Table 2.** The distribution range of valid sequences obtained by high-throughput sequencing in different sample types. **Supplementary Table 3.** Summary of alpha diversity indices calculated based on a cutoff of 97% similarity of 16S rRNA sequences. **Supplementary Figure 1.** Rarefaction analyses of OTUs clustered at 97% sequence identity of all samples: **(A)** Rarefaction curves on OTU level of the stomach contents and mucosae; **(B)** Shannon curves on OTU level of the stomach contents and mucosae; **(C)** Rarefaction curves on OTU level of the gill and intestinal samples; **(D)** Shannon curves on OTU level of the gill and intestinal samples. **Supplementary Figure 2.** Distribution of the bacterial communities in all samples at the phylum level: **(A)** Bacterial phyla in the stomach content and mucosa samples; **(B)** Bacterial phyla in the gill and intestinal samples. **Supplementary Figure 3.** Comparison of the bacterial phylum in the intestinal mucosae between the control and vaccinated groups. The significance of Welch t-test: ** *p* < 0.01. **Supplementary Figure 4.** Distribution of the bacterial communities in all samples at the genus level: **(A)** Bacterial genera in the stomach content and mucosa samples; **(B)** Bacterial genera in the gill and intestinal samples. **Supplementary Figure 5.** Comparison of the bacterial communities in the gill and stomach samples at the OTU level by LEfSe: **(A)** Comparison of the bacterial communities in the gill mucosae between the control and vaccinated groups; **(B)** Comparison of the bacterial communities in the stomach contents between the control and vaccinated groups; **(C)** Comparison of the bacterial communities in the stomach mucosae between the control and vaccinated groups. The highlighted taxa are enriched in the group that corresponds to each color; LDA scores can be interpreted as the degree of difference in the relative abundance of OTUs. **Supplementary Figure 6.** Metabolome data processing results: **(A)** Result of quality assurance; **(B)** Result of quality control (QC). The relative standard deviation (RSD) of the QC characteristic peak, that is, the coefficient of variation should not exceed 30%. **Supplementary Figure 7.** Principal component analysis (PCA) based on the Bray-Curtis distance visualizing the integral structure dissimilarities of microbial function.

**Additional file 2.** Supplementary Methods.

## Data Availability

The raw reads were deposited in the NCBI Sequence Read Archive under accession number PRJNA783994 (http://www.ncbi.nlm.nih.gov/sra).

## Abbreviations

*A. hydrophila*: *Aeromonas hydrophila*
*A. veronii*: *Aeromonas veronii*
NEW GIFT: NEW Genetically Improved Farmed Tilapia
GC-MS: Gas chromatography-mass spectrometry
COVID-19: Coronavirus disease 2019
TSA: Tryptic soy agar
PCR: Polymerase chain reaction
PBS: Phosphate buffer saline
EDTAK_2_: Ethylenediaminetetraacetic acid dipotassium
OTUs: Operational taxonomic units
LEfSe: Linear discriminant analysis effect size
LDA: Linear discriminant analysis
RBC: Red blood cells
WBC: White blood cells
Hb: Hemoglobin
PCV: Packed-cell volume
MCV: Mean corpuscular volume
MCH: Mean corpuscular hemoglobin
MCHC: Mean corpuscular hemoglobin concentration
RDW: Red blood cell distribution width
PLT: Platelet; MPV: mean platelet volume
PDW: Platelet distribution width
TP: Total protein
ALB: Albumin
GLO: Globulin
A/G: Albumin/globulin ratio
LZM: Lysozyme
SOD: Superoxide dismutase
