# Correction: Significant alterations of intestinal symbiotic microbiota induced by intraperitoneal vaccination mediate changes in intestinal metabolism of NEW Genetically Improved Farmed Tilapia (NEW GIFT, Oreochromis niloticus)

**DOI:** 10.1186/s40168-023-01464-7

**Published:** 2023-02-01

**Authors:** Zhenbing Wu, Qianqian Zhang, Jicheng Yang, Jinyong Zhang, Jie Fu, Chenyuan Dang, Mansen Liu, Shuyi Wang, Yaoyao Lin, Jingwen Hao, Meiqi Weng, Derong Xie, Aihua Li

**Affiliations:** 1 State Key Laboratory of Freshwater Ecology and Biotechnology, Institute of Hydrobiology, Chinese Academy of Sciences, Wuhan, 430072 China; 2 School of Environmental Science and Engineering, Huazhong University of Science and Technology, Wuhan, 430074 China; 3 Key Laboratory of Aquaculture Disease Control, Ministry of Agriculture, Beijing, China; 4 National Aquatic Biological Resource Center, NABRC, Wuhan, 430072 China; 5 College of Fisheries and Life, Dalian Ocean University, Dalian, 116023 China; 6 Laboratory of Aquatic Parasitology, School of Marine Science and Engineering, Qingdao Agricultural University, Qingdao, 266237 China; 7 College of Advanced Agricultural Sciences, University of Chinese Academy of Sciences, Beijing, 100049 China

**Correction: Microbiome 10, 221 (2022)**

**https://doi.org/10.1186/s40168-022-01409-6**

Following the publication of the original article [1], the author reported that the affiliation were incorrectly assigned to each authors. The affiliations are corrected above and the original article has been updated.
