# Supplementary material for: Significant alterations of intestinal symbiotic microbiota induced by intraperitoneal vaccination mediate changes in intestinal metabolism of NEW Genetically Improved Farmed Tilapia (NEW GIFT, Oreochromis niloticus)
Source: Microbiome. 2022 Dec 12;10:221. doi: 10.1186/s40168-022-01409-6 (PMC9742657; doi:10.1186/s40168-022-01409-6)
Supplement: Supplementary file 2 — Additional file 1: Supplementary Table 1. Results of tilapia body weight (kg) in different groups. Supplementary Table 2. The distribution range of valid sequences obtained by high-throughput sequencing in different sample types. Supplementary Table 3. Summary of alpha diversity indices calculated based on a cutoff of 97% similarity of 16S rRNA sequences. Supplementary Figure 1. Rarefaction analyses of OTUs clustered at 97% sequence identity of all samples: (A) Rarefaction curves on OTU level of the stomach contents and mucosae; (B) Shannon curves on OTU level of the stomach contents and mucosae; (C) Rarefaction curves on OTU level of the gill and intestinal samples; (D) Shannon curves on OTU level of the gill and intestinal samples. Supplementary Figure 2. Distribution of the bacterial communities in all samples at the phylum level: (A) Bacterial phyla in the stomach content and mucosa samples; (B) Bacterial phyla in the gill and intestinal samples. Supplementary Figure 3. Comparison of the bacterial phylum in the intestinal mucosae between the control and vaccinated groups. The significance of Welch t-test: ** p < 0.01. Supplementary Figure 4. Distribution of the bacterial communities in all samples at the genus level: (A) Bacterial genera in the stomach content and mucosa samples; (B) Bacterial genera in the gill and intestinal samples. Supplementary Figure 5. Comparison of the bacterial communities in the gill and stomach samples at the OTU level by LEfSe: (A) Comparison of the bacterial communities in the gill mucosae between the control and vaccinated groups; (B) Comparison of the bacterial communities in the stomach contents between the control and vaccinated groups; (C) Comparison of the bacterial communities in the stomach mucosae between the control and vaccinated groups. The highlighted taxa are enriched in the group that corresponds to each color; LDA scores can be interpreted as the degree of difference in the relative abundance of OTUs. Supplementary F [file 40168_2022_1409_MOESM1_ESM.pdf]

***Supplementary Data*:**

Title: Significant alterations of intestinal symbiotic microbiota induced by intraperitoneal vaccination mediate changes in intestinal metabolism of NEW Genetically Improved Farmed Tilapia (NEW GIFT, *Oreochromis niloticus*)

Supplementary Table 1. Results of tilapia body weight (kg) in different groups

**Initial group**	**Control group**	**Vaccinated group**
0.26	0.28	0.28
0.26	0.38	0.27
0.25	0.34	0.38
0.21	0.3	0.32
0.25	0.32	0.37
0.2	0.28	0.34
0.26	0.29	0.39
0.25	0.3	0.35
0.22	0.31	0.31
0.23	0.35	0.33
0.26	0.37	0.36
0.24	0.36	0.29
0.24 ± 0.02	0.32 ± 0.04 kg	0.33 ± 0.04 kg

Supplementary Table 2. The distribution range of valid sequences obtained by high-throughput sequencing in different sample types

Sample types	Number of valid reads per sample
Stomach contents (S)	3400-8286
Stomach mucosae (W)	2671-5471
Gill mucosae (G)	6393-10160
Intestinal contents (C)	5810-23908
Intestinal mucosae (M)	11980-70742

Supplementary Table 3. Summary of alpha diversity indices calculated based on a cutoff of 97% similarity of 16S rRNA sequences

**Group**	**Indices**				
	**OTUs**	**Chao**	**Shannon**	**Simpson**	**Coverage**
PS	252 ± 3.56	285.04 ± 11.30	4.54 ± 0.18	0.03 ± 0.01	98.17 ± 0.40%
VS	240 ± 9.87	270.74 ± 9.76	4.43 ± 0.25	0.03 ± 0.02	98.23 ± 0.28%
PW	180.75 ± 41.36	223.60 ± 48.92	3.62 ± 0.56	0.08 ± 0.05	98.14 ± 0.34%
VW	196.75 ± 52.21	236.08 ± 67.33	3.84 ± 0.56	0.06 ± 0.04	98.11 ± 0.56%
PG	53.75 ± 11.76	64.19 ± 14.68	2.07 ± 0.14	0.21 ± 0.03	99.81 ± 0.06%
VG	60 ± 3.56	68.14 ± 6.35	2.08 ± 0.06	0.22 ± 0.02	99.79 ± 0.04%
PC	32 ± 12.03	42.19 ± 16.61	1.14 ± 0.36	0.49 ± 0.16	99.87 ± 0.07%
VC	55.75 ± 23.29	69.34 ± 22.30	2.04 ± 0.92	0.28 ± 0.25	99.78 ± 0.05%
PM	54.5 ± 8.02	80.53 ± 30.31	1.45 ± 0.50	0.38 ± 0.14	99.78 ± 0.03%
VM	50 ± 5.35	55.28 ± 6.23	2.01 ± 0.25	0.24 ± 0.05	99.82 ± 0.04%

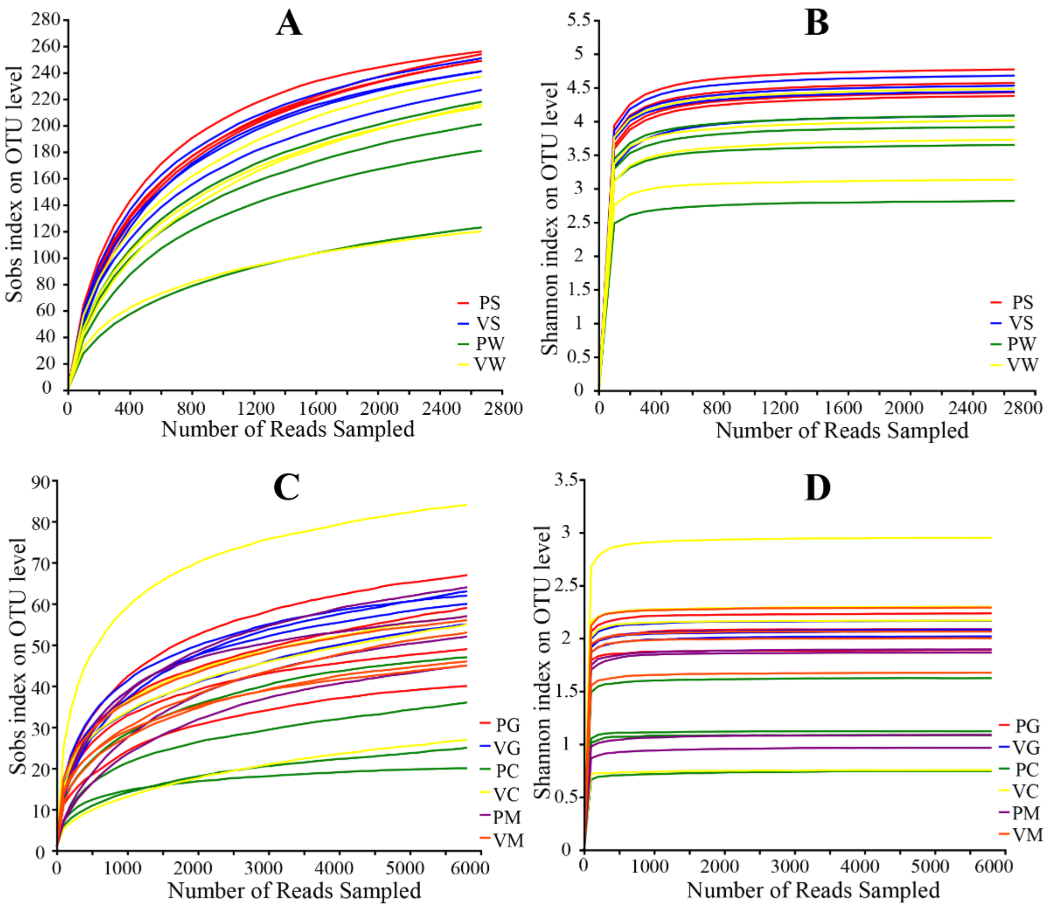

Supplementary Figure 1. Rarefaction analyses of OTUs clustered at 97% sequence identity of all samples: **(A)** Rarefaction curves on OTU level of the stomach contents and mucosae; **(B)** Shannon curves on OTU level of the stomach contents and mucosae; **(C)** Rarefaction curves on OTU level of the gill and intestinal samples; **(D)** Shannon curves on OTU level of the gill and intestinal samples. PS: stomach contents in the control group; VS: stomach contents in the vaccinated group; PW: stomach mucosae in the control group; VW: stomach mucosae in the vaccinated group; PG: gill mucosae in the control group; VG: gill mucosae in the vaccinated group; PC: intestinal contents in the control group; VC: intestinal contents in the vaccinated group; PM: intestinal mucosae in the control group; VM: intestinal mucosae in the vaccinated group.

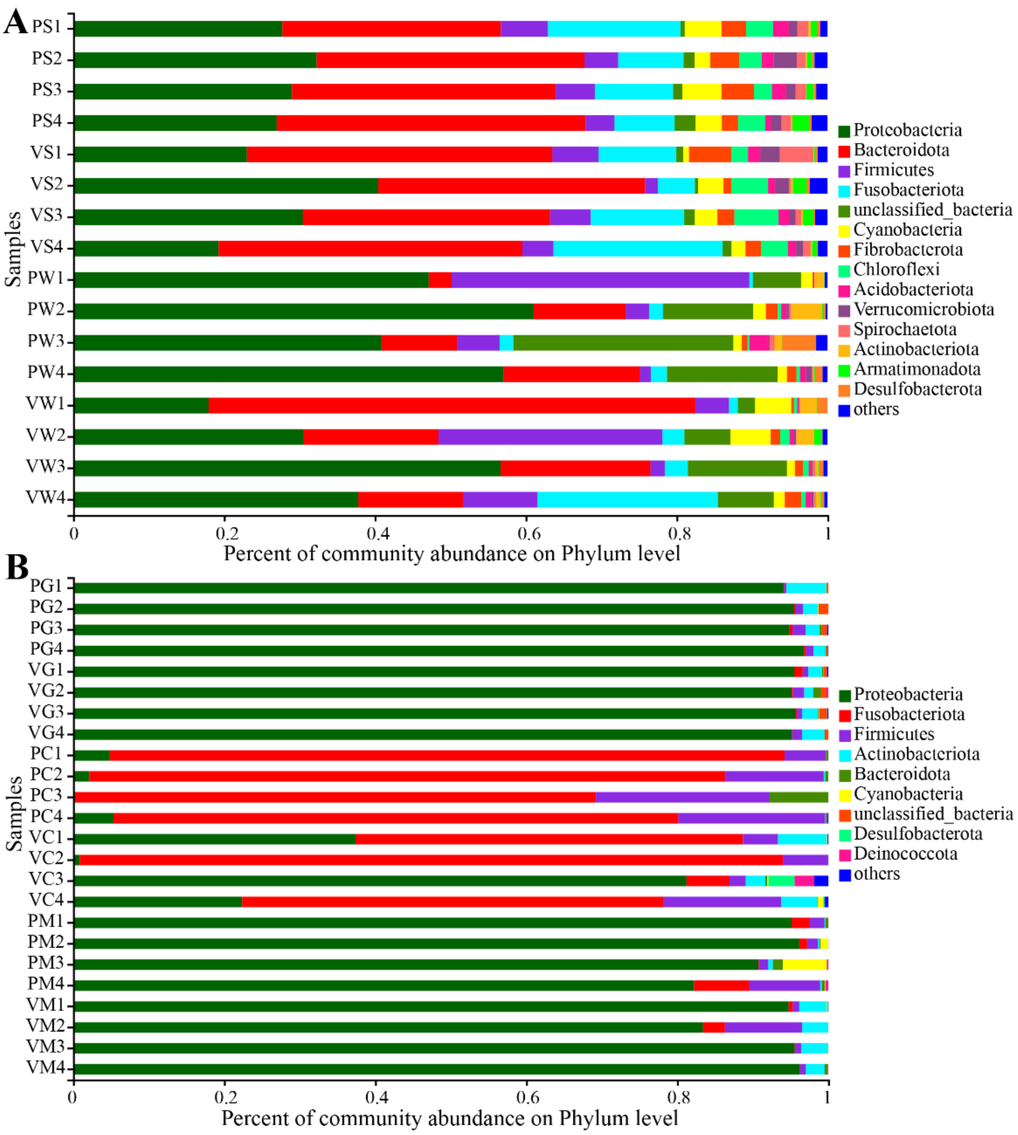

Supplementary Figure 2. Distribution of the bacterial communities in all samples at the phylum level: **(A)** Bacterial phyla in the stomach content and mucosa samples; **(B)** Bacterial phyla in the gill and intestinal samples. PS1-PS4: stomach contents in the control group; VS1-VS4: stomach contents in the vaccinated group; PW1-PW4: stomach mucosae in the control group; VW1-VW4: stomach mucosae in the vaccinated group; PG1-PG4: gill mucosae in the control group; VG1-VG4: gill mucosae in the vaccinated group; PC1-PC4: intestinal contents in the control group; VC1-VC4: intestinal contents in the vaccinated group; PM1-PM4: intestinal mucosae in the control group; VM1-VM4: intestinal mucosae in the vaccinated group.

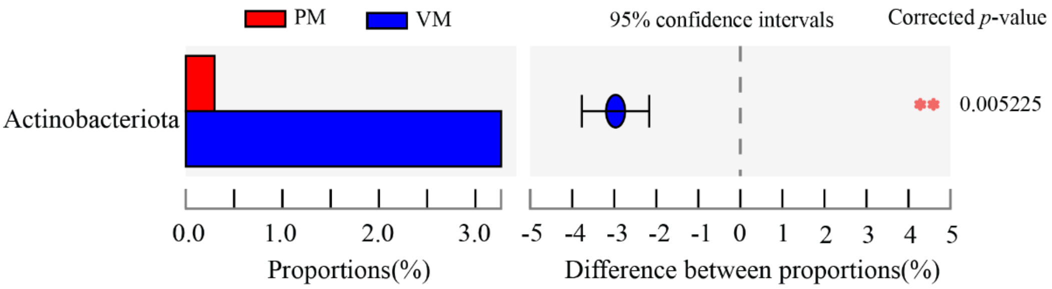

Supplementary Figure 3. Comparison of the bacterial phylum in the intestinal mucosae between the control and vaccinated groups. PM: intestinal mucosae in the control group; VM: intestinal mucosae in the vaccinated group. The significance of Welch t-test: ** $p < 0.01$.

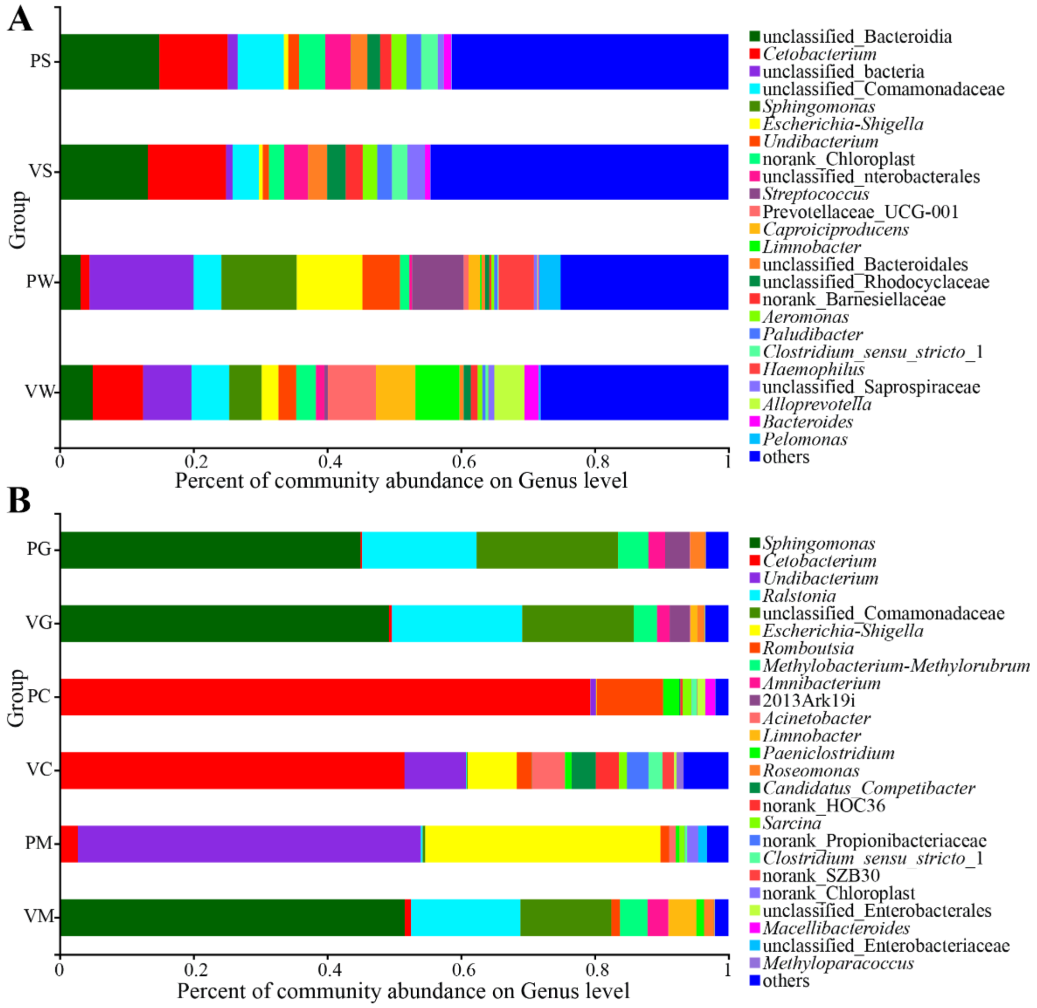

Supplementary Figure 4. Distribution of the bacterial communities in all samples at the genus level: **(A)** Bacterial genera in the stomach content and mucosa samples; **(B)** Bacterial genera in the gill and intestinal samples. PS1-PS4: stomach contents in the control group; VS1-VS4: stomach contents in the vaccinated group; PW1-PW4: stomach mucosae in the control group; VW1-VW4: stomach mucosae in the vaccinated group; PG1-PG4: gill mucosae in the control group; VG1-VG4: gill mucosae in the vaccinated group; PC1-PC4: intestinal contents in the control group; VC1-VC4: intestinal contents in the vaccinated group; PM1-PM4: intestinal mucosae in the control group; VM1-VM4: intestinal mucosae in the vaccinated group.

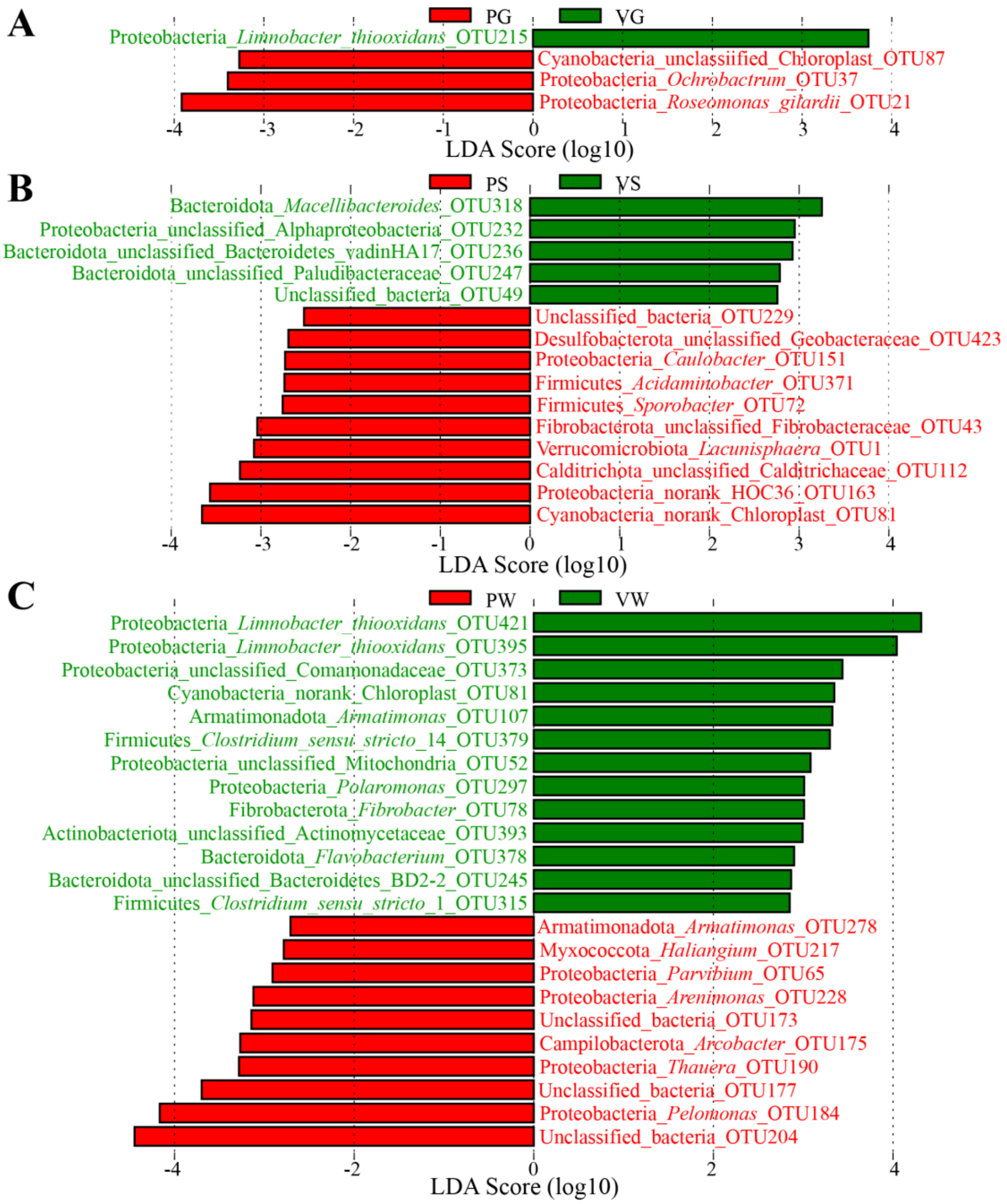

Supplementary Figure 5. Comparison of the bacterial communities in the gill and stomach samples at the OTU level by LEfSe: **(A)** Comparison of the bacterial communities in the gill mucosae between the control and vaccinated groups; **(B)** Comparison of the bacterial communities in the stomach contents between the control and vaccinated groups; **(C)** Comparison of the bacterial communities in the stomach mucosae between the control and vaccinated groups. The highlighted taxa are enriched in the group that

corresponds to each color; LDA scores can be interpreted as the degree of difference in the relative abundance of OTUs; PG: gill mucosae in the control group, VG: gill mucosae in the vaccinated group, PS: stomach contents in the control group, VS: stomach contents in the vaccinated group, PW: stomach mucosae in the control group, VW: stomach mucosae in the vaccinated group.

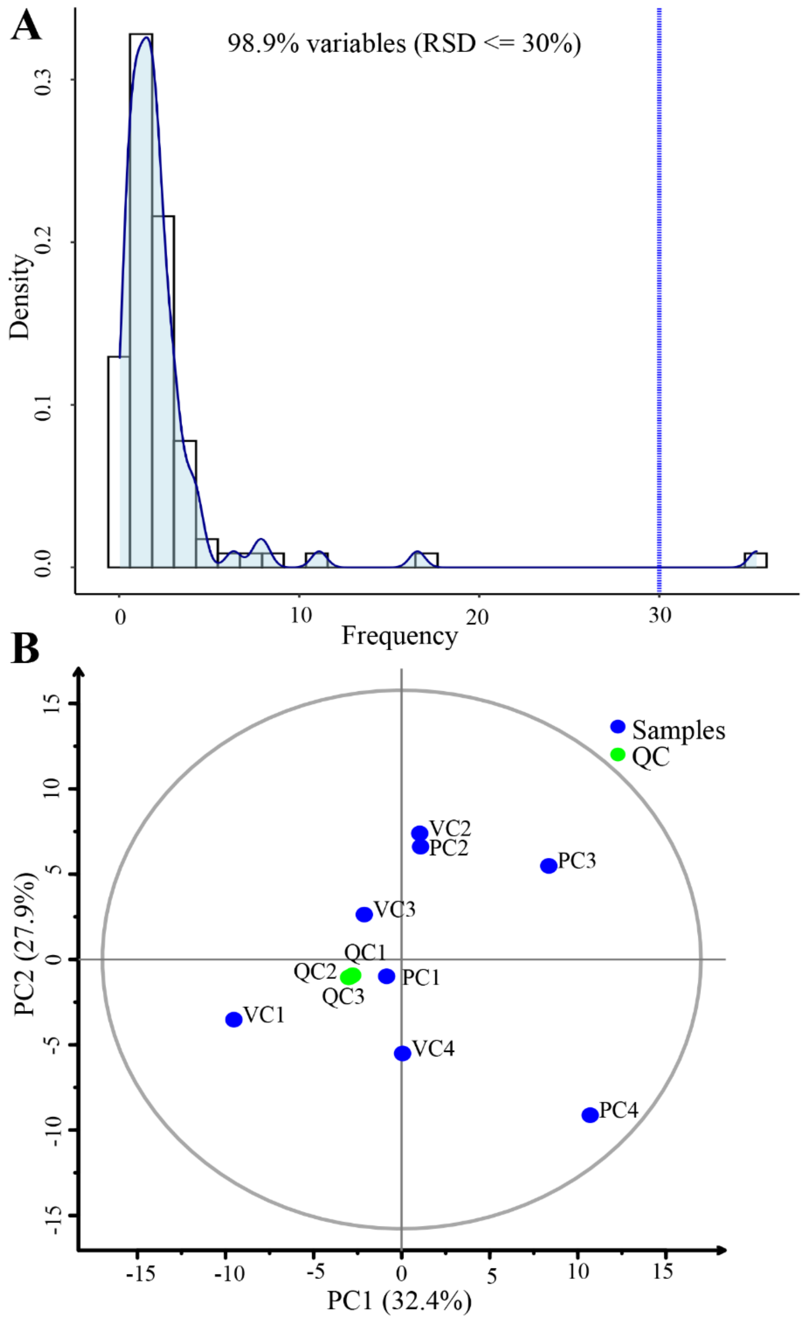

Supplementary Figure 6. Metabolome data processing results: **(A)** Result of quality assurance; **(B)** Result of quality control (QC). The relative standard deviation (RSD) of the QC characteristic peak, that is, the coefficient of variation should not exceed 30%.

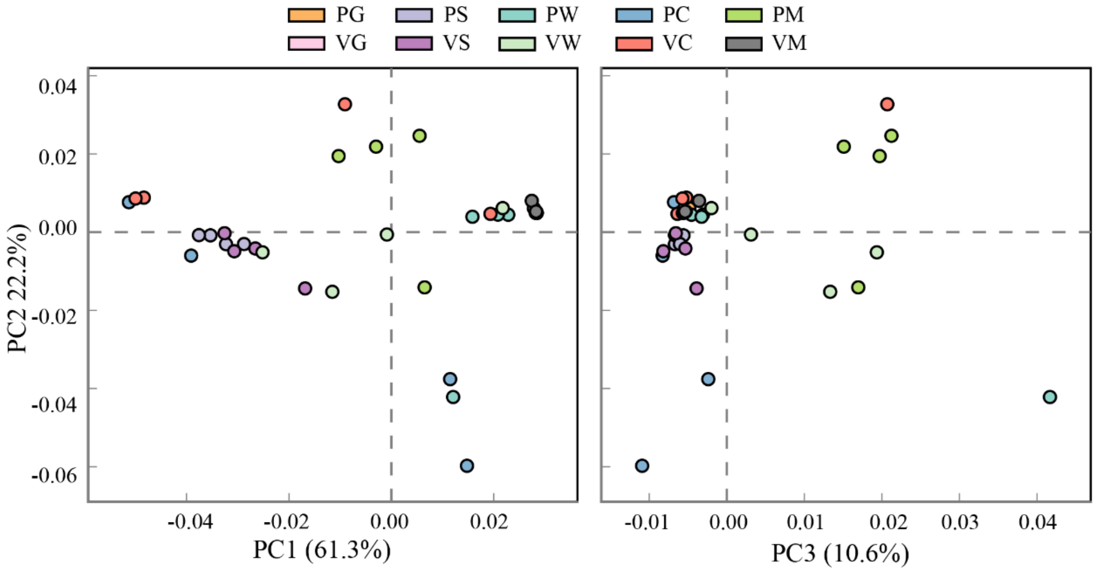

Supplementary Figure 7. Principal component analysis (PCA) based on the Bray-Curtis distance visualizing the integral structure dissimilarities of microbial function.
