# Supplementary material for: Significant alterations of intestinal symbiotic microbiota induced by intraperitoneal vaccination mediate changes in intestinal metabolism of NEW Genetically Improved Farmed Tilapia (NEW GIFT, Oreochromis niloticus)
Source: Microbiome. 2022 Dec 12;10:221. doi: 10.1186/s40168-022-01409-6 (PMC9742657; doi:10.1186/s40168-022-01409-6)
Supplement: Supplementary file 3 — Additional file 2. Supplementary Methods. [file 40168_2022_1409_MOESM2_ESM.pdf]

Supplementary Methods:

Metabolite extraction and detection from the intestinal content samples were performed as follows:

1 Preliminary and formal experiment processes [1, 2]:

1.1 Transfer 100 mg fecal samples into 5 mL centrifuge tubes, add 500 μL ddH_2O (4 °C), and vortex for 60 s;

1.2 Add 1,000 μL of methanol (pre-cooled at −20 °C), 60 μL of 2-Chloro-L-phenylalanine (0.2 mg/mL stock in methanol) and 60 μL of Heptadecanoic acid (0.2 mg/mL stock in methanol) as internal quantitative standard and vortex for 30 s;

1.3 Place the tubes into an ultrasound machine at room temperature for 10 min, and then stew for 30 min on the ice;

1.4 Centrifuge for 10 min at 12, 000 rpm 4 °C and 1.2 mL supernatant transfer into a new centrifuge tube. Samples were blow-dried by vacuum concentration;

1.5 Add 60 μL of 15 mg/mL methoxyamine pyridine solution, vortex for 30 s, and react for 120 min at 37 °C;

1.6 Add 60 μL BSTFA reagent (containing 1% TMCS) into the mixture, react for 90 min at 37 °C. Then, 12,000 rpm 4 °C centrifuges for 10 min and transfer the supernatant to inspect the bottle.

1.7 For the quality control (QC) samples, take 20 μL from each prepared sample extract and mix (These QC samples were used to monitor deviations of the analytical results from these pool mixtures and compare them to the errors caused by the analytical instrument itself) [3].

1.8 Use the rest of the samples for GC-MS test detection [4].

2 Gas chromatography-mass spectrometry conditions:

Gas chromatography was performed on an HP-5MS capillary column (5% phenyl/95% methylpolysiloxane 30 m × 250 μm i.d., 0.25 μm film thickness, Agilent J

& W Scientific, Folsom, CA, USA) to separate the derivatives at a constant flow of 1 mL/min helium. 1 μL of the sample was injected in the split mode in a 20:1 split ratio by the auto-sampler. The injection temperature was 280 °C, the interface was set to 150 °C, and the ion source was adjusted to 230 °C. The programs of temperature-rise were followed by an initial temperature of 60 °C for 2 min, 10 °C/min rate up to 300 °C and staying at 300 °C for 5 min. Mass spectrometry was determined by the full-scan method with a range from 35 to 750 (m/z) [1, 2].

3 Data pre-processing:

3.1 Raw gas chromatography/mass spectrometry (GC/MS) data were converted into the netCDF (network Common Data Form) format (namely XCMS input file format) via an Agilent MSD ChemStation workstation [5].

3.2 The XCMS (www.bioconductor.org; June 8, 2017) package in the R software (v3.1.3) was used to conduct peak identification, filtration, and alignment.

3.3 The data matrixes, including the mass-to-charge ratio (m/z), retention time, and intensity, were obtained. The annotation of metabolites using the Automatic Mass Spectral Deconvolution and Identification System (AMIDS) was searched against commercially available databases, such as the National Institute of Standards and Technology (NIST) and Wiley Registry Metabolomics Database. The alkane retention indices provided by the Golm Metabolome Database (GMD) (http://gmd.mpimpgolm.mpg.de/; June 8, 2017) were used for the further qualitative characterization of substances.

3.4 Most substances were further confirmed by the standard. The data were derived to Microsoft Excel (Microsoft, Redmond, WA, USA). Finally, the data were normalized to the internal standard for further statistical analyses.

Reference:

1. Ponnusamy K, Choi JN, Kim J, Lee SY, Lee CH. Microbial community and metabolomic comparison of irritable bowel syndrome faeces. J Med Microbiol. 2011;60(6):817-827.

2. Ng JSY, Ryan U, Trengove RD, Maker GL. Development of an untargeted metabolomics method for the analysis of human faecal samples using *Cryptosporidium*-infected samples. Mol Biochem Parasit. 2012;185(2):145-150.
3. Sangster T, Major H, Plumb R, Wilson AJ, Wilson ID. A pragmatic and readily implemented quality control strategy for HPLC-MS and GC-MS-based metabonomic analysis. Analyst. 2006;131(10):1075-1078.
4. Want EJ, Wilson ID, Gika H, Theodoridis G, Plumb RS, Shockcor J, et al. Global metabolic profiling procedures for urine using UPLC-MS. Nat Protoc. 2010;5(6):1005-1018.
5. Smith CA, Want EJ, O'Maille G, Abagyan R, Siuzdak G. XCMS: Processing mass spectrometry data for metabolite profiling using nonlinear peak alignment, matching, and identification. Anal Chem. 2006;78(3):779-787.
